# The Role of DNA Repair in Genomic Instability of Multiple Myeloma

**DOI:** 10.3390/ijms23105688

**Published:** 2022-05-19

**Authors:** Jana Yasser Hafez Ali, Amira Mohammed Fitieh, Ismail Hassan Ismail

**Affiliations:** 1 Biophysics Department, Faculty of Science, Cairo University, Giza 12613, Egypt; jali1@ualberta.ca (J.Y.H.A.); amohamed@sci.cu.edu.eg (A.M.F.); 2 Department of Oncology, Faculty of Medicine and Dentistry, University of Alberta, 11560 University Avenue, Edmonton, AB T6G 1Z2, Canada

**Keywords:** multiple myeloma, DNA repair, genomic instability, DNA damage response, myeloma therapeutics

## Abstract

Multiple Myeloma (MM) is a B cell malignancy marked by genomic instability that arises both through pathogenesis and during disease progression. Despite recent advances in therapy, MM remains incurable. Recently, it has been reported that DNA repair can influence genomic changes and drug resistance in MM. The dysregulation of DNA repair function may provide an alternative explanation for genomic instability observed in MM cells and in cells derived from MM patients. This review provides an overview of DNA repair pathways with a special focus on their involvement in MM and discusses the role they play in MM progression and drug resistance. This review highlights how unrepaired DNA damage due to aberrant DNA repair response in MM exacerbates genomic instability and chromosomal abnormalities, enabling MM progression and drug resistance.

## 1. Introduction

Multiple Myeloma (MM) is a B cell malignancy characterized by the neoplastic proliferation of long-lived plasma cells producing monoclonal immunoglobulins [1]. Long-lived plasma cells are terminally differentiated, non-dividing cells that survive for months/years in the bone marrow and produce antigen-specific immunoglobulins, thus forming an integral part of the immune defense system [1]. The affected plasma cells proliferate in the bone marrow resulting in extensive skeletal destruction with osteolytic lesions and fractures [2,3]. MM has a worldwide incidence of 160,000 cases a year with established risk factors such as age, male gender, familial and ethnic background, genetics, obesity, lifestyle, and environmental conditions [3].

MM arises from a pre-malignant, asymptomatic stage of plasma cell dyscrasia called Monoclonal Gammopathy of Unknown Significance (MGUS) [4,5]. Over time, MGUS can progress to asymptomatic Smoldering Multiple Myeloma (SMM) and then into overt and symptomatic Myeloma [1]. Both MGUS and SMM are differentially diagnosed by the amount of secreted monoclonal protein (M-protein) or extent of plasma cell (PC) involvement on bone marrow examination [5,6]. MGUS is primarily characterized by serum M-protein (IgM or non-IgM) < 30 g/L, urinary M-protein < 500 mg/24 h, clonal bone marrow PCs (BMPCs) < 10%, and a lack of end-organ damage, as per CRAB criteria [5]. SMM is diagnosed by serum M protein (IgG or IgA) levels of ≥30 g/L or urinary protein ≥ 500 mg/24 h and/or 10–60% clonal BMPCs [5]. Overt symptomatic MM is defined by the presence of a clonal PC population with at least one of the following myeloma-defining events (MDEs): (1) end organ damage attributable to plasma cell proliferation disorders as per CRAB criteria; (2) a biomarker of malignancy ≥ 1 [5]. The most typical clinical manifestations of MM have been summarized in the acronym CRAB: hyperCalcemia, Renal failure, Anemia and Bone damage [6]. The most common symptom at presentation is bone pain in the lumbar spine [5]. In diagnosing MM, a baseline metabolic panel should be performed to assess coagulation factors, renal and liver function, and serum analysis to obtain specific information about the type and quantities of M protein present [5]. Monoclonal immunoglobulins are markers of proliferative PC diseases and have been described extensively in the literature as serologic and urine-based tumor markers [7]. However, one major challenge is that their unique structures and molecular weight range (24–900 kDa) make it hard to develop a single test to diagnose or monitor all MM patients confidently [7]. As such, serum-free light chain ratio (sFLC), Serum Protein Electrophoresis (SPEP), and serum immunofixation electrophoresis (IFE) can detect 74%, 79%, and 87% of monoclonal antibodies, respectively, but all three tests combined can detect 98.6% of monoclonal antibodies [5].

## 2. MM Epidemiology and Pathophysiology

Genomic instability is a characteristic of most cancer cells, and MM is no exception. Genomic instability is the increased tendency of genomic alterations during cell division that results in base substitutions, insertions or deletions, copy number variations, and rearrangements due to chromosomal instability [8]. The pathogenesis of MM has been previously linked to the acquisition of genomic instability [8]. Historically, the use of fluorescence in situ hybridization (FISH) and cytogenetics has been for identifying chromosomal instability, including Immunoglobulin heavy chain (IgH) translocations and hyperdiploidy [9]. Half of the pre-malignant and clinical MM patients harbor one of two types of chromosomal abnormalities as the primary genetic lesion: Immunoglobulin heavy chain (IgH) translocations and hyperdiploidy [1]. Both types of genetic lesions involve large chromosomal structural events, which result in dysregulation of the G1/S cell cycle and cyclin D gene transcription [2].

Early in lymphopoiesis, B cells activate the V(D)J Recombination mechanism to form and edit antigen receptors by sequentially regulated DNA rearrangements of the IgH and IgL loci [10]. Later in the immune response, IgH class switch recombination (CSR) and somatic hypermutations (SHM) occur during antigen-driven differentiation of B lymphocytes [11]. IgH translocations are mediated primarily by errors in IgH switch recombination, resulting in gene enhancer elements that lead to the overexpression of oncogenes such as MMSET/FGFR3, CCND3, CCND1, MAF, and MAFB [12]. Oncogene dysregulation by primary IgH translocations increases proliferation and/or inhibits differentiation [13].

Hyperdiploidy is characterized by trisomies of odd-numbered chromosomes [12]. These initial genetic events are followed by secondary genetic events that include the gain of genetic material (i.e., chromosome 1q) and deletion of parts of the chromosome (i.e., 1p, 13q, and 17p) [12]. The development of MM is reflected in the progressive increase in the acquisition of secondary genomic alterations such as copy number abnormalities, DNA hypermethylation, and several somatic mutations that affect DNA repair pathways, RNA metabolism, and NF-kB and MAPK pathways [14].

MM may also be characterized through molecular marker-driven classification systems, including gene expression profiling [15]. MM patient samples have identified novel driver mutations in transcription factors BRAF, FAM46C, DIS3, XBP1, IRF4, and PRDM1, as well as various histone-modifying enzymes MLL, MLL2, MLL3, UTX, MMSET (WHSC1), WHSC1L1, and recent inference toward HOXA-9 [15]. Understanding the precise genome maintenance mechanisms that initiate and dictate disease progression is essential to our understanding of the pathogenesis of MM and the development of novel therapeutic strategies. Although genomic instability is a common cause of myelomagenisis, it also creates a vulnerability to be exploited for treatment, which will be discussed later in the review. Despite our current understanding of clinical subtypes and disease biology, several diagnostic challenges stand in the way between patient care and disease progression.

## 3. DNA Repair Pathways in MM

As mentioned earlier, genomic instability is a well-recognized hallmark of cancer development and progression, and MM is no exception [8]. MM cells present genomic instability for which its molecular basis is not fully understood. Every day, cells are faced with thousands of DNA lesions that must be repaired to preserve the function and survival of different organisms [16]. DNA Damage Response (DDR) involves a complex network of proteins that detects and repair many DNA insults [17].

After DNA damage occurs, DNA repair proteins identify the damage sites and determine whether and how to repair the damage depending on the type of lesion or push the cells towards apoptosis (programmed cell death) or halt the cell cycle until the repair is completed through DNA damage checkpoints [18]. Mammalian cells are equipped with several DNA repair pathways, including base excision repair (BER), nucleotide excision repair (NER), mismatch repair (MMR), inter cross-link repair pathway, non-homologous end-joining (NHEJ), and homologous recombination (HR) to repair DNA damage. In the Section 4, we will provide an overview of DNA Repair Pathways, focusing on how they profoundly impact MM genomic instability and treatment; for a summarized look at the results, please refer to Table 1.

### 3.1. Base Excision Repair (BER) Pathway

The BER pathway is a cellular mechanism that repairs DNA lesions caused by oxidation, deamination, depurination, and alkylation [42,43]. Four enzymatic steps are essential for the function of the BER pathway: DNA glycosylase, AP endonuclease, DNA polymerase, and DNA ligase. In the initiation step of BER, a damage-specific DNA glycosylase identifies and removes the damaged base from DNA [42]. DNA glycosylases are either mono- or bi-functional depending on which bonds are cleaved during the damage removal process [42]. Monofunctional glycosylases perform base excision only using an activated water molecule for nucleophilic attacks on the N-glycosidic bond.

In contrast, bifunctional glycosylases use an amino group of a lysine side chain for the same purpose, forming a Schiff-base intermediate and subsequently cleaving DNA backbone 3′ to the lesion [42]. Additionally, DNA glycosylases are sorted into four superfamilies based on their structural characteristics and have different targets with redundancy in oxidative lesions, which exemplifies the biological importance of repairing said lesions [44]. Two families of DNA glycosylases are relevant to our discussion: the Uracil DNA Glycosylases (UDG) superfamily and the helix-hairpin-helix (hHh) glycosylases [45].

**Figure 1:**
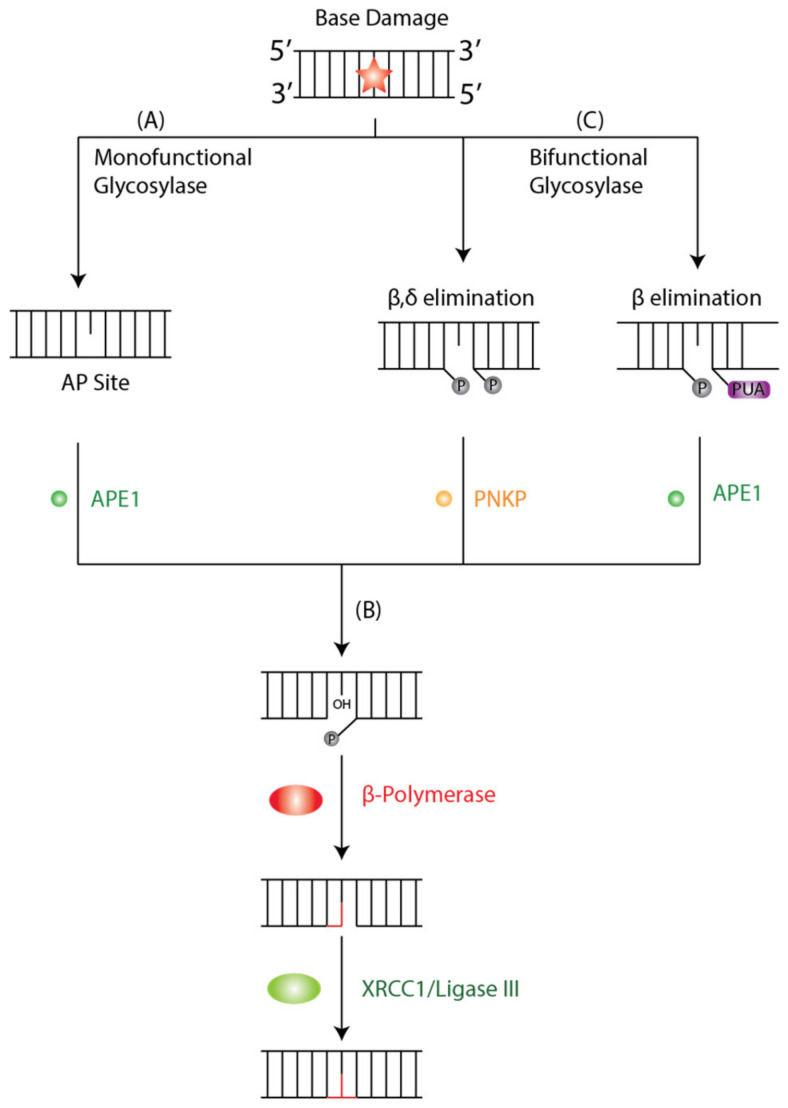
Schematic Representation of BER Pathway. BER is initiated by DNA Glycosylases that recognize and bind to a base lesion. (**A**) Monofunctional Glycosylases will identify the DNA lesion and catalyze the hydrolysis of the N-glycosyl bond that releases the damaged base and generates an AP site that is processed by APE1. (**B**) A bifunctional glycosylase will recognize and remove oxidative lesions either through β,δ or β-elimination to create a single strand break. The 3′ α,β-unsaturated aldehyde and 5′ phosphate are further processed by PNKP and APE1, respectively. (**C**) Subsequently, Polβ and XRCC1/Ligase III fill and seal the single nucleotide gap and restore the original base sequence. The orange star denotes base damage.

A monofunctional DNA glycosylase such as Thymine DNA Glycosylase (TDG) or MutY homolog DNA glycosylase (MUTYH) will produce an apurinic/apyrimidinic (AP) intermediate site by removing the damaged base by cleaving the N-glycosidic bond between the target base and deoxyribose (Figure 1A). The AP site is further processed by AP-endonuclease 1 (APE1), which creates a single-stranded DNA nick 5′ to the AP site and a single nucleotide gap in the DNA, causing a 3′-hydroxyl and 5′ phosphate. The polymerase β lyase domain removes the 5′-phosphate terminal, and a new nucleotide is inserted into the gap by Polβ’s transferase activity (Figure 1B). Finally, the nick is sealed by XRCC1/Ligase III, and the lesion is repaired. In contrast, a bifunctional glycosylase such as 8-OxoG DNA glycosylase 1 (OGG1) exhibits both glycosylase and AP-lyase activity where it will initiate the BER cycle by removing the damaged base and cleaving DNA at the 3′ phosphodiester bond of the 3′ AP site by β or β,δ-elimination to create a single-stranded break (Figure 1C). The 3′ α,β-unsaturated aldehyde and 5′ phosphate groups are further processed by APE1 and PNKP, respectively. Both APE1 and PNKP cleave a hydroxyl at the 3′-terminus allowing for subsequent gap filling by β-Polymerase (Polβ) and ligation by XRCC1/Ligase III, as described in classical BER.

Previous studies have linked abnormalities in BER-related genes and proteins to neurological disorders such as Alzheimer’s [46] and Huntington’s disease [47], cancer, and other diseases such as asthma [48] and ischemia/reperfusion (I/R) events [49]. In MM cell lines, very little data exist to implicate BER in MM. One study used retrospective analysis, genotyping SNPs, and arrayed primer extension (APEX) technology in 169 patients receiving high dose melphalan for MM found a polymorphism in APE1 or MUTYH represent independent prognostic factors significantly associated with the shorter survival of MM patients [19]. Another study looked at 93 MM patients and 192 controls to evaluate the impact of polymorphisms in genes encoding the four main proteins of the BER system: OGG1, XRCC1, APE1, and MUTYH on the risk and survival of MM [19]. This study found that the OGG1 genotype associated with low DNA repair type had an increased risk of MM, with no statistically significant differences in the risk of MM in the allele and genotype frequencies of the XRCC1, APE1, and MUTYH polymorphisms. These data suggested that low BER activity might induce higher levels of DNA damage and induce malignant transformation of the plasma cell and progression to aggressive myeloma [19]. Other studies have reported a range of methylation densities of TDG in several MM cell lines but not in normal human plasma cells [20]. The hypermethylation of TDG in MM cell lines leads to lower gene expression levels, resulting in less efficient DNA repair activity in response to hydrogen peroxide-induced DNA damage, which can be rescued by the expression of exogenous TDG. Other in vitro and in vivo studies support the involvement of TDG in cancer by showing that TDG regulates the expression of tumor suppressor genes by interacting with several transcription factors [42].

### 3.2. Nucleotide Excision Repair (NER) Pathway

The NER pathway is a DNA repair mechanism that removes a broad range of base lesions from the genome, such as photoproducts generated by UV radiation, base adducts created by chemical agents such as melphalan, cisplatin, or benzopyrene, and base lesions produced by reactive oxygen species (ROS) [50]. Two subpathways can initiate NER: global genome NER (GG-NER) or transcription-coupled NER (TC-NER) [51]. The entire genome is examined in the GG-NER pathway for helix distortions associated with structural changes to nucleotides. In contrast, TC-NER is activated when RNA polymerase II (RNA Pol II) is stalled during transcription in the template strand.

**Figure 2:**
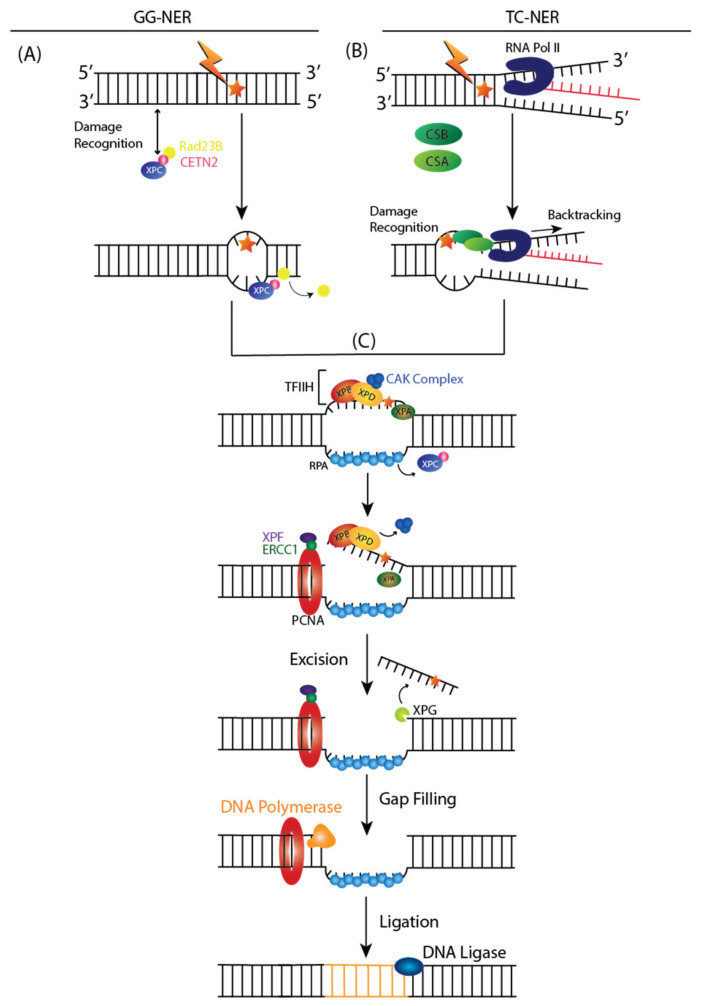
Nucleotide Excision Repair (NER) Subpathways: (**A**) Global Genome NER (GG-NER) and (**B**) Transcription-Coupled NER (TC-NER). (**A**) Helix distorting lesions are recognized with the help of XPC, Rad23B, and Centrin2 (CETN2). After XPC binds to the damage, Rad32B dissociates from the complex. (**B**) Damage is indirectly recognized by stalled RNA Pol II at the site of the lesion. CSB-CSA complex is formed, resulting in RNA Pol II backtracking to increase the accessibility of DNA lesions for repair. (**C**) In both GG-NER and TC-NER, the TFIIH complex is recruited post-lesion recognition. Upon binding of TFIIH, the CAK complex will dissociate from the core of the TFIIH, and the double helix further opens up around the lesion. XPD, XPA, and XPB will verify the existence of the lesions and binds to the single-stranded chemically altered nucleotide. RPA then coats the undamaged strand while XPF-ERCC1 heterodimer will create a 5′ incision to the lesion. Then, XPG is activated and will cut the damaged strand 3′ to the lesion, which excises the strand. PCNA will recruit DNA polymerases for gap-filling DNA synthesis and is finally sealed by DNA Ligase 1 or 3. The orange star denotes DNA damage.

In GG-NER, XPC and UV excision repair proteins RAD23 homolog B (RAD23B) and Centrin 2 (CETN2) recognize DNA damage by binding to destabilized helical structures of DNA (Figure 2A) [51]. Upon binding of XPC complex to DNA lesion, RAD23B dissociates from the complex [51].

TC-NER indirectly detects DNA damage by sensing lesion-stalled RNA Pol II, which recruits ERCC8 or Cockayne Syndrome Protein A (CSA) and ERCC6 or Cockayne Syndrome Protein B (CSB) (Figure 2B) [51]. Upon RNA, Pol II is stalled at the DNA lesion, and a CSA-CSB complex is formed, resulting in RNA Pol II backtracking and rendering the DNA accessible for repair [51].

After damage recognition, Transcription Initiation Factor IIH (TFIIH) is recruited to the lesion in both GG and TC NER, and the CDK Activating Kinase (CAK) subcomplex dissociates from the core of TFIIH (Figure 2C) [51]. XPD and XPB, two helicase subunits, open the DNA around the lesion [51]. During this step, Replicating Protein A (RPA) is recruited to single strand undamaged DNA. XPA recruits a structure-specific endonuclease, XPF-ERCC1 heterodimer, and is directed to the damaged strand to create an incision 5′ to the lesion [51]. XPG is activated and cuts the damaged strand 3′ to the lesion, which excises the lesion within a 22–30 nucleotide-long strand, which triggers a DNA-damage signaling reaction [51]. Finally, a new DNA is synthesized by DNA polymerases, proliferating cell nuclear antigen (PCNA), and replication factor C (RFC), using the intact strand as a template and terminated by XXRC1/ligase III [51].

Abnormalities in NER-related genes have been described in several malignancies with potential implications for clinical outcomes [22]. The literature has previously reported epigenetic hypermethylation of the hHR23B gene, which codes for RAD32B in response to DNA damage in IL-6 responsive MM KAS-6/1 cells, which is absent from normal resting human B cells [21]. Due to the NER pathway’s role in p53 dependent apoptosis, the epigenetic silencing of hHR23B is hypothesized to favor the clonal expansion of MM cells [21]. Additionally, one study evaluated NER activity in 20 MM cell lines. It showed that the expression of canonical NER gene ERCC3 significantly impacted the outcome in newly diagnosed MM patients when treated with alkylating agents [22]. They also demonstrated that targeting XPB leads to NER inhibition, which significantly increased sensitivity to melphalan. In line with this evidence, another study found that patients with a combination of ERCC2 K751Q and XRCC3 T241M had the best prognosis defined as time to treatment failure due to suboptimal DNA repair, which increases sensitivity to treatment [25]. Similarly, another study found that, in a cohort of 187 MM patients, those with SNPs in ERCC5 (rs1047768) and XPA (rs1800975) had more prolonged overall survival after autologous stem-cell transplantations [23].

### 3.3. Mismatch Repair (MMR) Pathway

MMR is critical for maintaining genomic stability by targeting base substitutions and insertion-deletion mismatches that arise during replication and recombination [52]. Four genes regulate the MMR mechanism: MutL Homologue 1 (MLH1), MutS Homologue 2 (MSH2), MutS Homologue 6 (MSH6), and PMS2 that code for proteins that function as heterodimers. In vitro studies showed the mammalian MMR mechanisms involve the following steps: lesion recognition, repair initiation, lesion excision, and DNA resynthesis [53,54].

MMR is initiated when a mismatch is detected, MSH2 associates with either MSH6 or MSH3 forming MutSα and MutSβ, respectively, and MSH1 couples with PMS2, PMS1, or MLH3, forming MutLα, MutLβ, and MutLγ, respectively [55]. MutS recognizes mismatched base pairs, insertions/deletions of 1–4 nucleotides in one strand, and contains a weak ATPase activity, which may play a role in both mismatch recognition and signaling other proteins to assemble in the mismatch repair complex. MutL is needed for the interaction between MutS and MutH [56]. MutSα is primarily responsible for repairing single base-base insertion and deletion loops (IDL) mismatches, while MutSβ is responsible for repairing IDL mismatches containing up to 16 extra nucleotides in one strand [57]. MutLα is a latent endonuclease that introduces a 5′ and 3′ nick into the daughter strand in an ATP-dependent manner and in conjunction with other MMR proteins, which activates downstream pathways leading to mismatch removal [58]. MutLγ is an endonuclease that nicks DNA in a MutLβ and loop-dependent manner [59]. Recent studies have implicated mammalian MutLα and MutLγ proteins in triplet repeat DNA expansion, which causes neurological disorders such as Fragile X syndrome [60].

**Figure 3:**
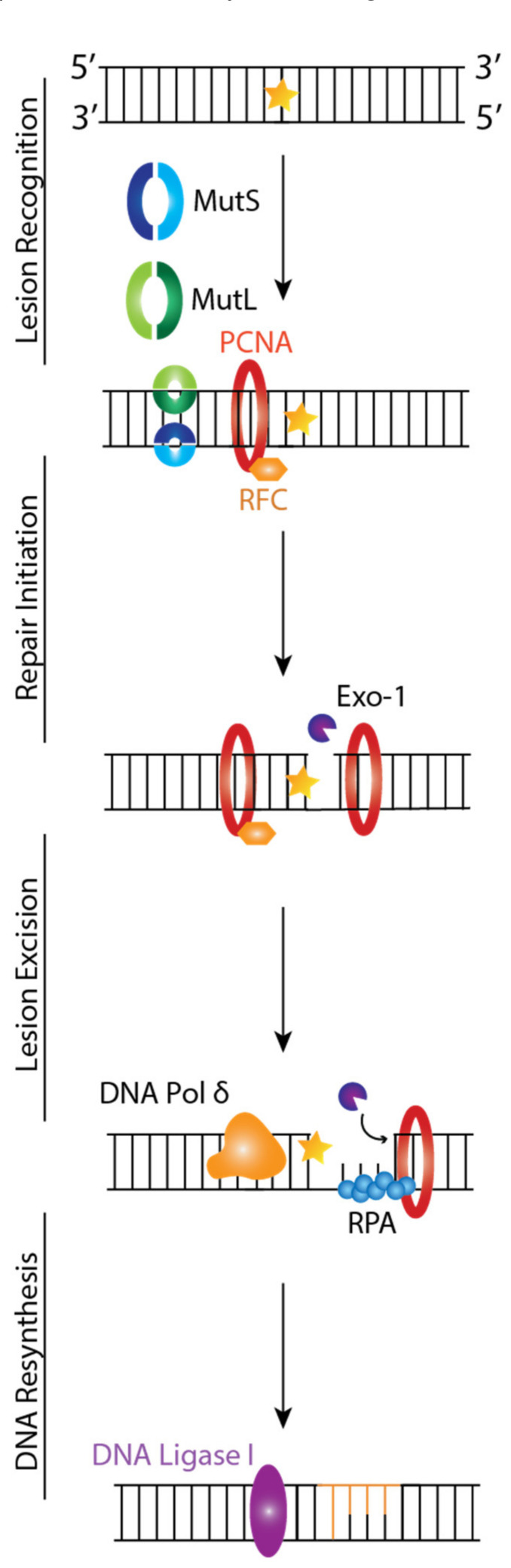
Mismatch Repair (MMR) Pathway. MutS and MutL start DNA repair by recognizing and binding to mismatches. PCNA and RFC will be recruited to the site of damage to initiate repair. These interactions will lead to the recruitment of Exo1 to a strand break where it will excise the damaged DNA from the nick up to and beyond the mismatch depending on the MutS, MutL, and RPA. During DNA resynthesis, DNA Pol δ will use the parental strand as a template to repair the mismatch, followed by DNA Lig I ligation. The orange star denotes DNA mismatch.

The MutS and MutL complex will first recognize the DNA lesion by binding to the mismatch while checking post-replicated DNA (Figure 3) [53]. This triggers interactions and communications between MSH proteins, proliferating cell nuclear antigen (PCNA), Replication Factor C (RFC), and MutLα (Figure 3). These interactions lead to the recruitment of Exonuclease 1 (EXO1) to a strand break located in the newly synthesized DNA, where it will carry out DNA excision from the nick up to and beyond the mismatch dependent on MutSα/β, MutLα, and Replication Protein A (RPA) (Figure 3). Finally, DNA Polymerase δ will carry out DNA resynthesis by using the parental DNA strand as a template, followed by DNA Ligase I-catalyzed nick ligation.

Microsatellite Instability (MSI) is a manifestation of defective MMR and is found in 50% of patients with MM [61]. Microsatellites are polymorphic repetitive DNA sequences distributed along with coding and non-coding regions of the genome [55]. The repetitive nature of the sequences makes them sensitive to mismatch errors, and the accumulation of mutations of repeat length alterations is defined as MSI. In line with this evidence, a microsatellite analysis showed instability in one or more of 9 loci found in 15 of 92 patients: 20.7% of MM/plasma cell leukemia (PCL), 12.5% of relapsed MM/PCL, and 7.7% of MGUS/SMM [28]. Additionally, another study found that methylation-mediated silencing is a frequent event in monoclonal gammopathies: 83% of MM, 46% of MGUS, and 77% of plasmacytomas have at least one gene methylated, affecting different molecular pathways involved in cell cycle, DNA repair, and apoptosis [26]. In terms of MMR, hMLH1 was hypermethylated in 10% of MM cases and unmethylated in all instances of MGUS and plasmacytomas, and this is associated with poorer survival [26,27]. This evidence shows that MMR defects are found in plasma cell dyscrasias. The increased frequency during more active stages of the disease (MM > Plasmacytomas > MGUS) suggests a contributory role in the malignant transformation of plasma cells [27,62].

Additionally, the MMR and BER pathways are required for the processing of activation-induced cytidine deaminase (AID)-indued uracil lesions into DSBs that are necessary for Ig CSR and SHM [42]. An increasing body of evidence suggests that aberrant targeting of AID contributes to point mutations and translocations of oncogenes associated with B cell malignancies such as MM [62]. One study analyzed whole genomes of SMM patients progressing into MM and found that all SMM and MM samples were characterized by an early and major contribution from AID [41]. These results represent an early and common driver mutational process, consistent with AID activity in the germinal center and its absence in MM cells [41,63].

### 3.4. Inter Cross-Link Repair Fanconi Anaemia (FA) Pathway

FA is a genetic disease characterized by bone marrow failure, cancer predisposition, and genomic instability [64]. Thus far, 19 FA genes have been identified (FANCA to FANCT); any inactivation in these genes is associated with the FA phenotype. The proteins encoded by the 19 FA genes coordinate different steps in repairing interstrand crosslinks (ICLs). ICLs are DNA lesions that inhibit replication and transcription, and they must be repaired to ensure cell survival. The pathway for DNA repair includes multiple steps, including lesion recognition, DNA incision, lesion bypass, and lesion repair.

**Figure 4:**
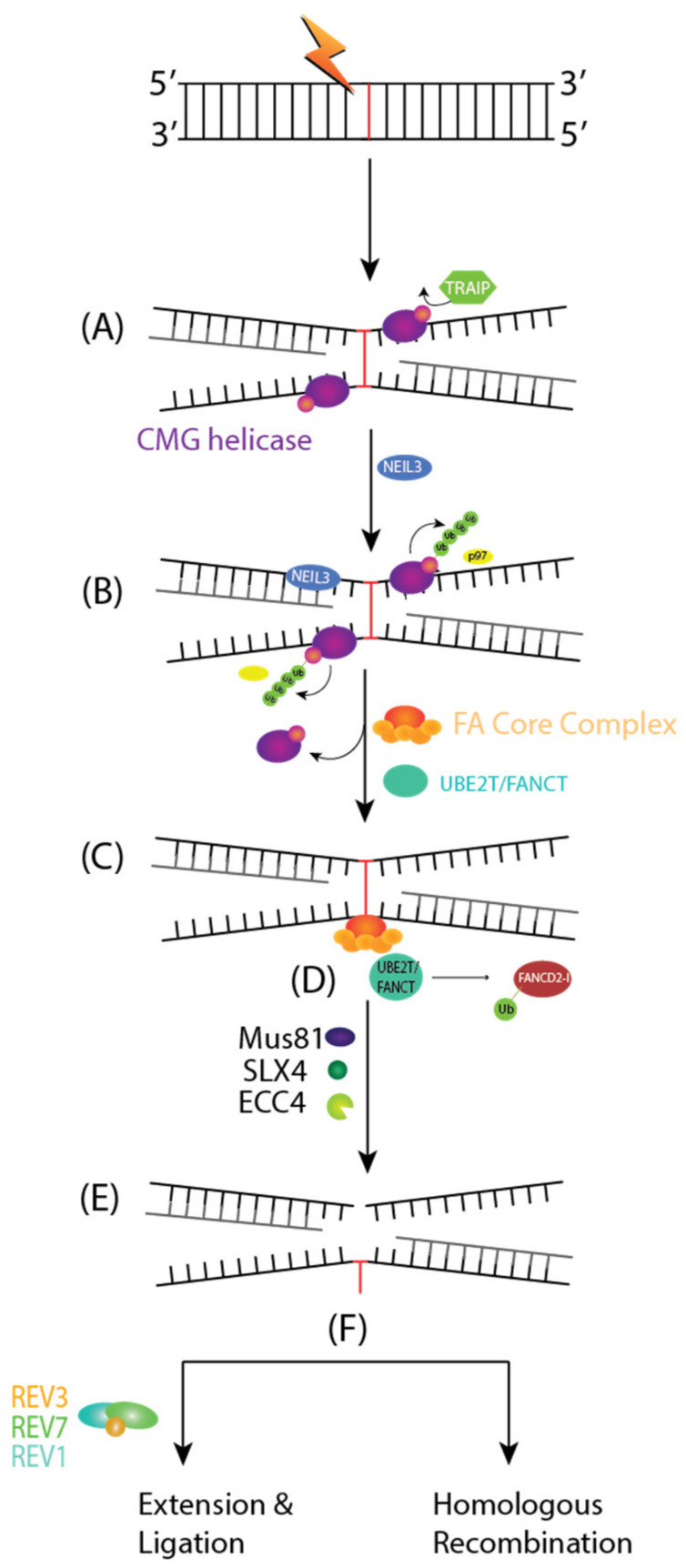
Fanconi Anemia (FA) Pathway of Interstrand Crosslinks (ICLs). (**A**) ICLs occurring during the S phase of the cell cycle will converge two replication forks creating an X-shaped structure surrounding it. TRAIP will ubiquitinate CMG helicase to recruit NEIL3 Glycosylase for an incision-dependent unhooking mechanism of ICL resolution. (**B**) Subsequently, CMG is evicted from the chromatin through long Ub chains and p97, allowing for the approach of both replication forks towards the ICL. This commits the repair to FA pathway-mediated ICL repair. (**C**) The FA Core Complex is recruited to the chromatin through UHRF1 and FANCM-MHF1-MHF2 complex (not depicted). (**D**) The FA Core Complex and UBE2T/FANCT E2 conjugating enzymes will monoubiquitinate FANCD2-I. FANCD2-I will recruit Mus81, SLX4, and ERCC4 endonucleases (**E**) to cleave the DNA strand contiguous to the ICL and generate a DNA adduct and an ICL-derived DSB. (**F**) The DNA adduct is bypassed by REV1, REV7/FANCV, and REV3 and a DSB will be repaired through HR, respectively.

The FA pathway of DNA repair is activated when DNA replication forks are stalled and they encounter an ICL in the S phase of the cell cycle. Any ICLs occurring outside of the S phase are repaired through NER. ICL repair starts with the convergence of two replication forks, creating an X-shaped DNA structure surrounding ICLs. A CDC45-MCM-GINs or CMG helicase complex is ubiquitinated by a TRAIP, an E3 Ubiquitin Ligase (Figure 4A). The short ubiquitin chains recruit NEIL3 glycosylase for an incision-dependent unhooking mechanism for ICL resolution. Then, CMG is evicted from the chromatin using long ubiquitin chains and p97, allowing for the approach of both replication forks towards the ICL and committing toward the FA pathway mediated ICL repair (Figure 4B) [65].

ICLs are detected by a UHRF1 protein and the FANCM-MHF1-MHF2 complex, which, respectively, recruits the FANCD2-I heterodimer and the FA core complex to chromatin (Figure 4C) [66]. The FA core complex is an E3 Ubiquitin Ligase made of 10 FA proteins: FANCA, FANCB, FANCC, FANCE, FANCF, FANCG, FANCL, FAAP100, FAAP20, and FAAP24. The FA core complex and UBE2T/FANCT E2 conjugating enzyme will monoubiquitinate FANCD2-I (Figure 4D). The ubiquitylated FANCD2-I recruits SLX4/FANCP, a scaffolding protein for the endonucleases Mus81, SLX1, and XPF/ERCC4/FANCQ. These endonucleases will cleave the DNA strand contiguous to the ICL and generate a DNA adduct and an ICL derived DSB (Figure 4E). The former is bypassed by the REV1, REV7/FANCV, and REV3 translesion synthesis complex, while the latter is repaired by HR (Figure 4F).

The FA pathway ensures the fidelity of the ICL-derived DSB by activating the ATR-CHK1 cell cycle checkpoint and ensuring DNA repair through the high-fidelity homologous recombination pathway. Refer to the Section 4.3 of this paper for a more detailed description of the HR pathway starting at the end resection step.

Both FA and MM share some clinical similarities such as hematological complications and pancytopenia, anemia, and bone marrow failure because of DNA damage repair deficiency [66,67]. As mentioned earlier, the FA pathway is regulated through the monoubiquitylation of FANCD2 [68]. Downstream FA pathway components and associated factors such as SLX4 exhibit ubiquitin-binding motifs that are important for their DNA repair function [68]. Ubiquitin plays a role in cellular protein turnover, including tagging proteins for degradation through the proteosome [68]. Previous studies have shown that proteasome function is required for the activation of the FA pathway and DNA damage signaling [69]. The deregulation of proteasome function disrupts the normal degradation process, which results in a buildup of proteins within the cell, eventually causing toxicity and cell death [69]. As such, proteasome inhibitors have been historically used to induce toxicity towards proliferating malignant cells due to their therapeutic index [69]. In the case of MM, proteosome inhibitors such as PS-341 have been previously reported to sensitize tumor cells relative to DNA damaging agents such as melphalan and cisplatin [70]. One study showed that inhibiting proteosomes using Bortezomib and MG132 or depleting proteasome subunits 19S and 20S inhibited the monoubiquitylation and nuclear formation of FANCD2 and DNA damage signaling players such as Phospho-ATM, 53BP1, NBS1, BRCA1, and RAD51 [38]. The inhibition of both the FA pathway and DDR may explain the sensitization of tumor cells to DNA damaging agents induced by proteasome inhibitors.

Additionally, many other FA-associated MM abnormalities have been reported in the literature. Neri and Bahlis observed a significant downregulation of DNA glycosylases such as UNG2, NEIL1, and MP in MM cells resistant to melphalan, which was associated with increased efficiency of single strand breaks (SSBs) or DSBs [71]. Whole exon sequencing of human MM cell lines reported alterations in 54% of FA genes, FANCI, FANCA, FANCD2, and BRCA, related to MM patients at relapse [39]. RNAseq analysis showed an increased expression of genes associated with DNA repair such as FANCI, FANB, RAD51, and BRCA1 in MM patient samples [39]. Previous studies have shown that the FA pathway contributes to acquired drug resistance in melphalan-resistant myeloma cell lines such as 8226/LR5 and U266/LR5 [71], and the disruption of this pathway reverses drug resistance [36].

Previous studies have shown that proteasome function is required for the activation of the FA pathway and DNA damage signaling, such as FANCD2 and SLX4 [38]. Therefore, inhibiting proteasome functions with PS-341 has been previously reported to sensitize tumor cells to DNA damaging agents such as melphalan and cisplatin [70]. Furthermore, inhibiting proteosomes using Bortezomib and MG132 or depleting proteasome subunits 19S and 20S inhibited monoubiquitylation and nuclear formation of FANCD2 and DNA damage signaling players such as Phospho-ATM, 53BP1, NBS1, BRCA1, and RAD51 [38]. The inhibition of both the FA pathway and DDR may explain the sensitization of tumor cells to DNA damaging agents induced by proteasome inhibitors. This sensitization was also reported as a bortezomib-induced “BRCAness” that sensitizes MM cell lines and primary CD138^+^ cells to poly ADP ribose polymerase (PARP) inhibitors and confirmed in MM xenografts in SCID mice [33].

## 4. Repair of DNA Double-Strand Breaks (DSBs)

DSBs are lesions formed when both DNA strands are broken. Of the various DNA damage insults, DSBs are considered the most significant type of damage due to their cell toxicity. DSBs can arise following exposure to (programmed or spontaneous) endogenous or exogenous agents. There are two main pathways for repairing DSBs: Non-Homologous End Joining (NHEJ) and Homologous Recombination (HR).

### 4.1. Canonical Non-Homologous End Joining (c-NHEJ)

**Figure 5:**
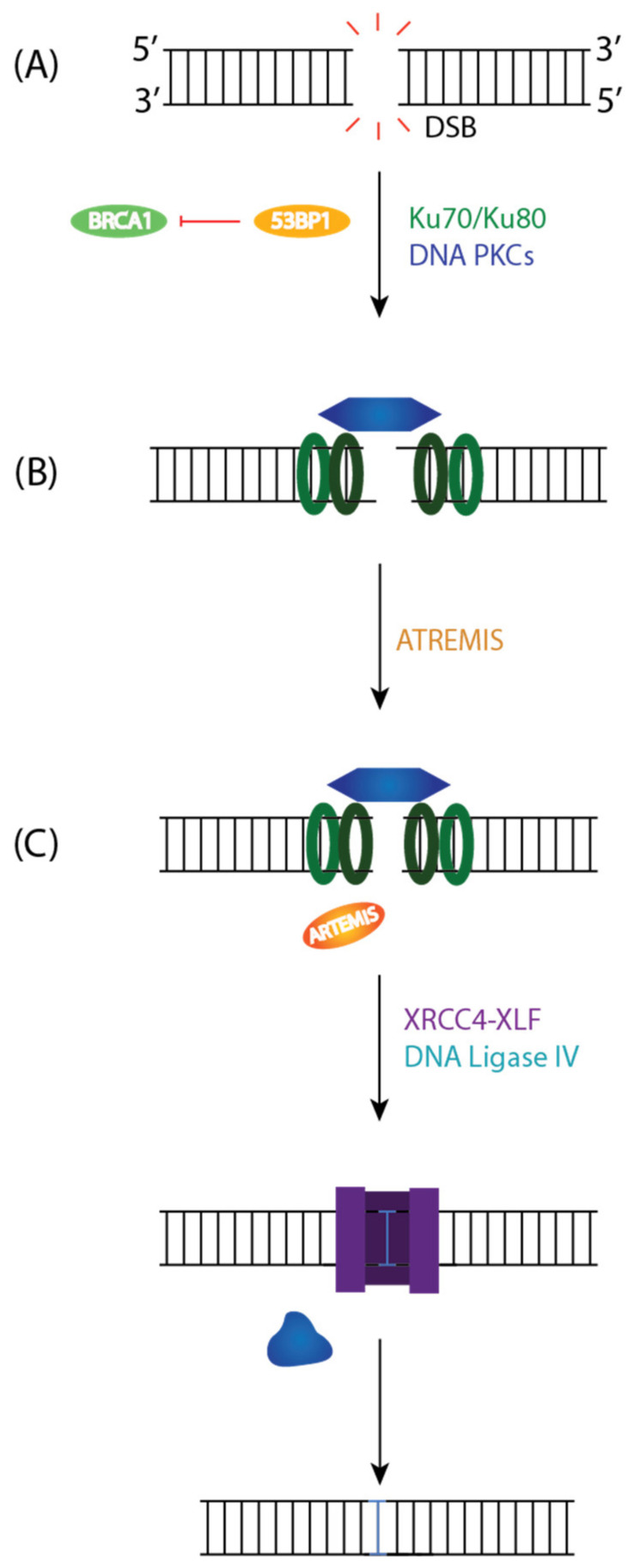
Canonical Non-Homologous End Joining (c-NHEJ) Pathway to Repair DSBs. (**A**) 53BP1 directs the repair pathway to NHEJ by inhibiting BRCA1 and end resection. The Ku80/Ku70 heterodimer binds to the DNA ends, aligns them, and recruits DNA PKcs to the DSB to activate its kinase domain. (**B**) DNA PKcs is autophosphorylated and will activate ARTEMIS resulting in limited resection. (**C**) The Ligase Complex, DNA Lig IV, and XRCC4 will stabilize the positioning of the ends before restoring the break.

c-NHEJ repair is active in all cell cycle phases and can directly ligate broken DNA ends regardless of sequence. DNA ends are recognized by Ku70 and Ku80 (XRCC4) heterodimer, followed by the recruitment and activation of DNA-dependent protein kinase catalytic subunit (DNA-PKcs). If the ends are chemically incompatible or damaged, nucleases such as ARTEMIS can trim the ends while XRCC4-DNA Ligase IV (LIG4) seals the break [72]. More importantly, c-NHEJ is directly involved in the relegation step of DSB introduced by RAG endonucleases during VDJ recombination during Ig isotype switching in B cells [73]. VDJ recombination is a site-specific recombination process that occurs early in the development of B and T lymphocytes [74]. It induces site-specific DSBs several times in order to generate antigen receptor diversity during the development of each new lymphocyte [75]. Despite the critical importance of this pathway in generating antigen receptor diversity, errors that occur during VDJ recombination can contribute to genomic instability and the development of lymphoma and leukemia [75].

c-NHEJ is the primary pathway for repairing DNA DSBs throughout the cell cycle [76]. Following DNA Damage, 53BP1 is phosphorylated by ATM, which inhibits DNA end resection (an initial step in HR that results in 5′–3′ degradation to generate a long 3′ single-stranded DNA overhangs) and BRCA1, which allows for the retention of Ku on DNA ends (Figure 5A) [77]. DNA ends are recognized by Ku70-Ku80 heterodimer (Ku), which will recruit and activate DNA-PKcs. DNA-PKcs is recruited to DSB through Ku and form the DNA-PK complex (Figure 5A). Most DSBs have two incompatible ends that will block direct ligation. As a result, nucleases such as Artemis will trim the ends by degrading short regions of the 5′ or 3′ overhangs to generate small regions of microhomology between strands that can facilitate end joining (Figure 5B). Artemis is an endonuclease with a conserved Metallo-β-lactamase and β-CASP domain with 5′-exonuclease activity [77]. The DNA-PKcs and Artemis complex have endonuclease activity on the 5′ and 3′ DNA overhangs at duplex ends. While Artemis is thought to be the primary nuclease, other nucleases such as MRE11, CtBP Interacting Protein (CTIP), Werner syndrome ATP-dependent helicase (WRN), Flap endonuclease 1 (FEN1), and EXO1 can contribute to the repair of the DSBs [78]. The final step of c-NHEJ is ligating broken ends by DNA ligase IV and dissolution of the NHEJ complex (Figure 5C) [76]. XRCC4-XLF complex forms a sleeve-like structure around a DNA duplex that will stabilize the positioning of the ends before covalent ligation.

In the absence of c-NHEJ repair, alternative NHEJ (alt-NHEJ) can occur [79], alt-NHEJ is a repair process that is homology-independent [80], independent of Lig IV, and can recognize DNA ends using different DNA Pol (δ and θ) and DNA Nucleases (ERCC1-XPF) and Ligases (Lig I and Lig III/XRCC1) [81]. There is an increasing interest in this pathway in malignant cells, as it induces genomic rearrangements such as large deletions and translocations [79].

**Figure 6:**
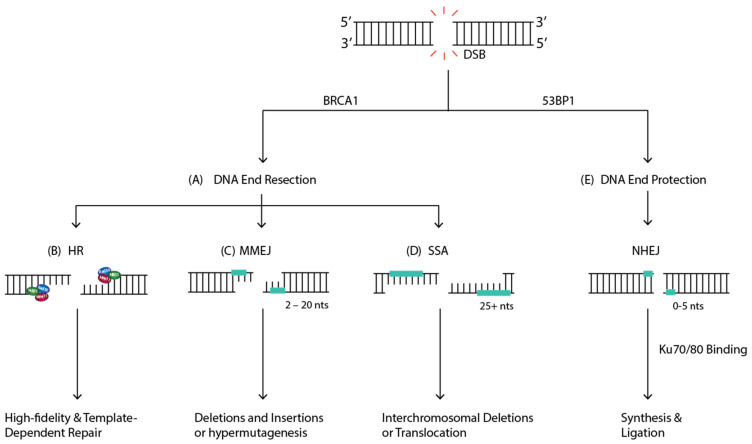
The Different Outcomes of DSB Repair. When a DSB forms, one of two things can occur, (**A**) DNA end resection can dictate pathway choice. If DNA ends are resected, one of three pathways can be used to repair the lesion: Homologous Recombination (HR), Single-Strand Annealing (SSA), or Microhomology-mediated End Joining (MMEJ). (**B**) HR repair will continue if the cells are in S/G2 phase and will result in high-fidelity and template dependent DSB repair. If there is no sister chromatid available in the cell, one of two pathways can be used for repair: MMEJ or SSA. (**C**) MMEJ involves microhomology, ranging from 2 to 20 nucleotides in length and can ultimately result in genomic instability in the form of deletions and insertions in chromosomes or hypermutagenesis. (**D**) SSA requires end resection and complementary sequences more than 25 nucleotides in length and can generate interchromosomal deletions or translocations. (**E**) If the DNA ends are protected, canonical NHEJ (c-NHEJ) will dominate where Ku70/80 heterodimer will bind, resulting in minimal end processing, synthesis, and ligation.

In mammalian cells, the repair of DSBs by alt-NHEJ is more evident in the absence of a functional NHEJ pathway [79]. Based on the amount of DNA sequence homology used to align DNA ends, the alt-NHEJ pathway is mediated by two minor pathways: microhomology-mediated end joining (MMEJ) and single-strand annealing (SSA) [82]. MMEJ involves microhomology sequences that range from 2 to 20 nucleotides long (Figure 6C) [82]. MMEJ can induce genomic instability through deletions and insertions at the breakpoint junctions and hypermutagenesis at the flanking DNA sequence [83]. On the other hand, in SSA, 5′ to 3′ end resection at both ends exposes single-strand regions complementary repeat sequences that are more than 25 nucleotides long (Figure 6C) [82]. The complementary sequences anneal and generate a DNA duplex with non-complementary 3′ ssDNA tails. The tails are removed, followed by gap-filling synthesis and ligation, usually generating intrachromosomal deletions or translocations [82].

Compared to other repair pathways, the factors involved in alt-NHEJ are not well defined, so we will focus on canonical NHEJ phenotypes in MM. Several reports have documented contradicting results on the NHEJ phenotype in MM cell lines. One study used clonogenic survival and functional repair assays and concluded that MM cell lines RPMI 8226, OPM2, and U266 exhibited corrupt NHEJ repair [73]. Contrastingly, another group found that NHEJ efficiency increased in MM cells [84]. A Western blot analysis of seven MM cell lines, three LINF control cell lines, and HeLa cells revealed higher expression of DNA PKcs in six out of seven MM cell lines than in controls [84]. Other readouts, including XRCC4 and DNA Ligase IIIα, show clearly higher expression than controls, suggesting upregulated c-NHEJ and alt-NHEJ, respectively [84]. Additionally, NHEJ efficiency was measured using an extrachromosomal assay where end joining is determined by measuring cells’ ability to recircularize an enzyme-digested plasmid and corroborated by an integrated intrachromosomal substrate that was integrated into the chromatin of U266, JJN3, and control LINF cell lines [84]. They found that NHEJ efficiency was significantly higher in MM compared to the control LINF cell lines [84]. Previously described DNA Ligase IV (*LIG4*) polymorphism, *LIG4 T9I*, was significantly associated with a two-fold reduction in developing MM [34]. The polymorphism is not thought to affect the stability of DNA Ligase IV. Still, it is predicted to increase the hydrophobicity of the region tertiary structures and alter the protein–protein interactions with other components of the NHEJ pathway. These data suggest that the functional variation of NHEJ because of LIG4 polymorphism can modulate the risk of MM. However, a recent study with a larger sample size did not find an association between either SNP or the risk of MM [85]. The same study reported an association between LIG4 rs1555902. It decreased MM risk, which approached statistical significance. There is a significant association between Activation Induced Cytidine Deaminase (AICDA)-rs3794318 and better outcome [85]. AICDA is an enzyme that initiates the CSR process by creating U-G mismatches that Uracil DNA Glycosylases or substrates can excise for AP endonucleases [85]. When two nicks originated this way and are close to each other, DSBs can arise spontaneously or through the intervention of MMR and BER repair pathways [85]. Hayden et al., genotyped 27 haplotype tagging SNPs in XRCC4 and XRCC5 and found preliminary evidence of an association between the polymorphisms and myeloma [35]. The study speculates that SNP XRCC4 rs963248 affects the stability of the XRCC4 mRNA transcript or alters its expression. Additionally, the XRCC5 rs105168 can result in exon skipping and errors in alternative splicing patterns that affect mRNA stability and translation [35].

### 4.2. VDJ Recombination

VDJ recombination is the somatic recombination that occurs in developing lymphocytes during the early stages of T and B cell maturation, resulting in a highly diverse repertoire of antibodies and antigen receptors [75]. This process describes how lymphocytes recombine a repertoire of germline Variable (V), Diversity (D), and Joining (J) exon gene segments in various permutations to generate antigen receptor diversity in immunoglobulins and T cell receptors. In short, VDJ recombination introduces DSB adjacent to a pair of segments, deletes or inverts the intervening DNA, and then ligates the segments together.

**Figure 7:**
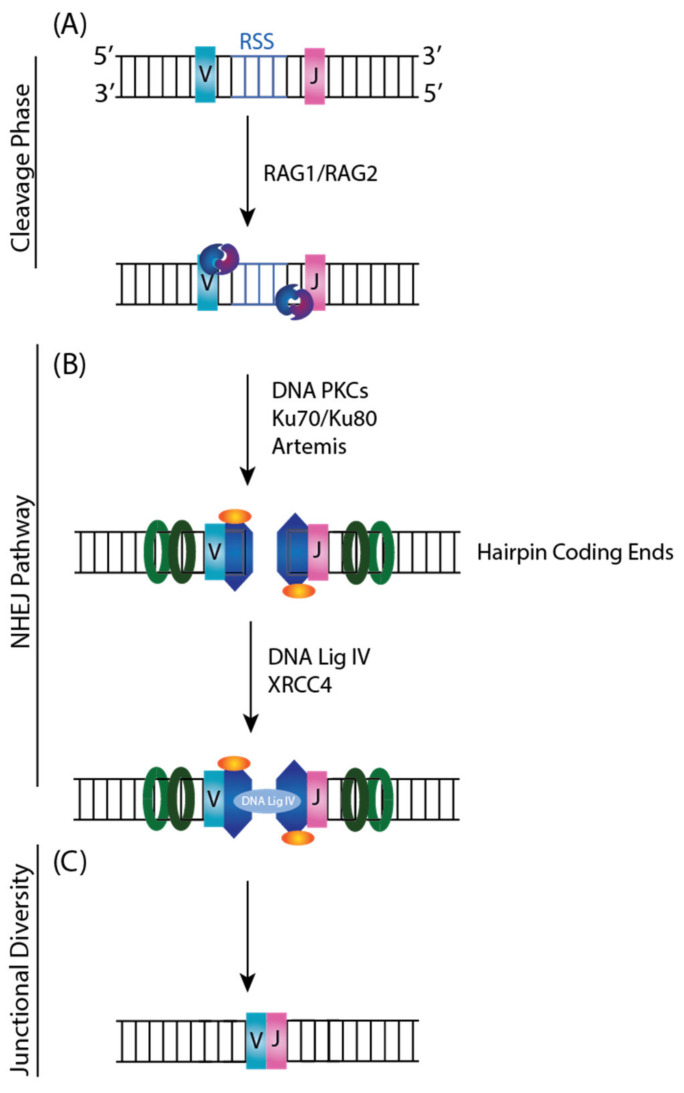
V(D)J Recombination in Lymphocytes and NHEJ. (**A**) RAG1 and RAG2 lymphoid-specific proteins recognize RSS located adjacent to V, D, and J coding segments. RAG1 and RAG2 initiate the cleavage phase and introduce a pair of DSBs between the RSS and the V, D, and J coding segments resulting in hairpin coding ends or blunt phosphorylated ends (not depicted). (**B**) NHEJ machinery will then recognize the hairpin coding end and repair RAG generated breaks to (**C**) create a diverse repertoire of antibodies and antigen receptors.

The VDJ Recombinase consists of two lymphoid-specific proteins, RAG1 and RAG2, that recognize conserved DNA sequence elements called Recombination Signal Sequences (RSS) (Figure 7A). RSSs are located adjacent to each V, D, and J coding segment. They are composed of a conserved palindromic heptamer and an AT-rich nonamer sequence separated by non-conserved 12 or 23 base pair spacers [79]. The orientation of the RSS will dictate whether the reaction proceeds with an inversion or deletion [75]. RAG1 and RAG2 initiate the cleavage phase by recognizing the RSSs and introducing a pair of DSBs between the RSS and V, D, and J coding segments resulting in two types of ends: coding hairpin ends, and blunt 5′-phosphorylated signal ends. Then, NHEJ machinery such as Ku heterodimer, DNA-PKcs, MRE11, and DNA Ligase IV-XRCC4 will recognize and repair the RAG generated breaks (Figure 7B) [86]. The ability to rearrange germ-line DNA to generate diversity is an essential prerequisite for a functional immune system. However, it represents one of the processes that can occasionally lead to the malignant transformation of cells by translocation of protooncogenes into immunoglobulin loci.

Consistent with the role that NHEJ plays in VDJ recombination, previous studies have shown that the inactivation of DNA-PKcs leads to immune deficiency [87,88]. Additionally, NHEJ proteins have been implicated more broadly in accelerated tumor development in aggressive Pro-B cell lymphomas due to the misrepair of DSBs generated during antigen receptor generation [89]. One example is that Ku-deficient cells and mice have increased genomic instability, including chromosomal breakage, translocations, and aneuploidy [90]. In addition, Ku deficient mice display defects in VDJ recombination [91] and B/T cell maturation [90]. In MM, switch regions have been shown to mediate translocations in a subset of MM patients, suggesting a role in transformation [92]. These translocations typically resulted in dysregulation and the increased expression of oncogenes that are positioned near one or more strong IGH, IGK, and IGL enhancers [93]. Translocations mediated by VDJ recombination will result in an enhancer relocation to one or more derivative chromosomes such as der(14) for IGH, der(non-2) for IGK, and der(non-22) for IGL. These data provide insight into how the dysregulation of DNA repair at the damage sites in MM may underpin the characteristic genomic instability of the disease. Ultimately, more research can be directed towards characterizing this link between the Ig rearrangements in MM and NHEJ to understand the impact of treatment on malignant clones and provide specific targets to monitor the tumor cells.

### 4.3. Homologous Recombination (HR)

**Figure 8:**
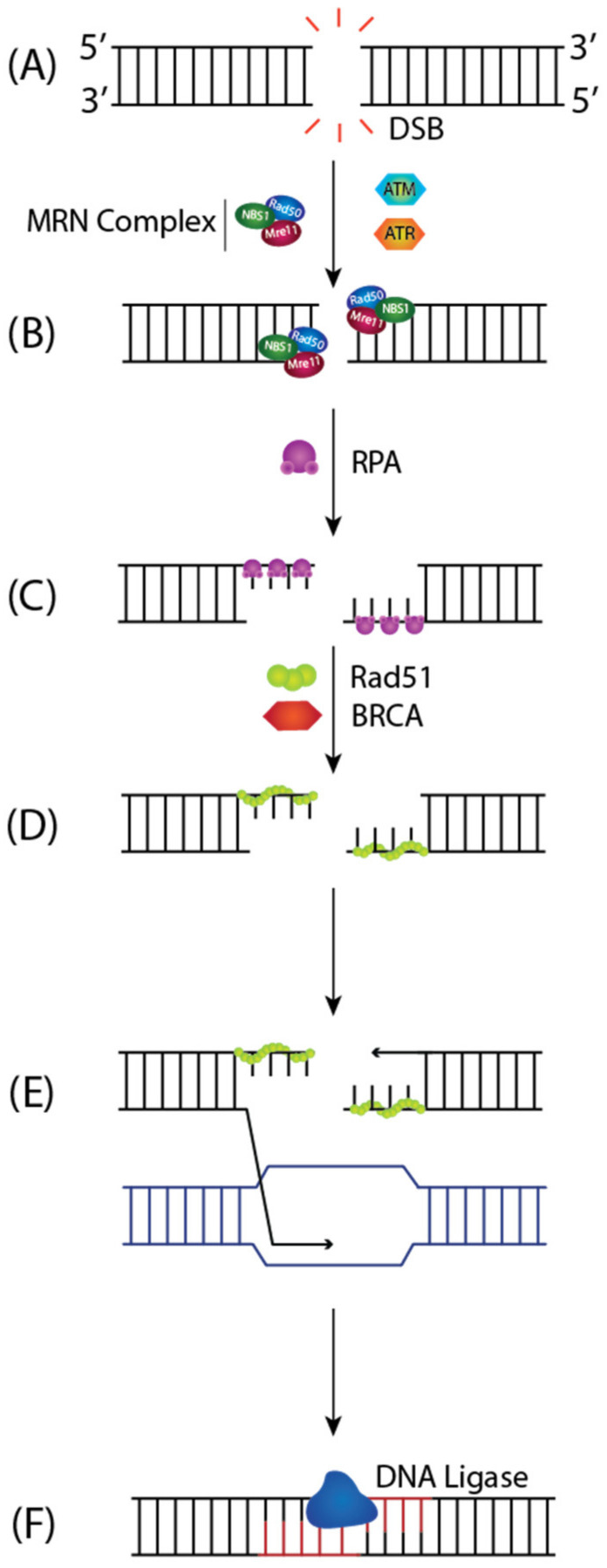
Homologous Recombination (HR) Repair of DSBs. (**A**) HR begins with the 5′-3′ extensive resection of break ends by MRN complex to (**B**) generate 3′ ssDNA overhangs that are coated with RPA. Once the DNA ends are captured, ATM and ATR kinases trigger downstream signaling to facilitate DNA repair through HR. (**C**) Rad51 is then loaded onto the ssDNA in a BRCA-dependent manner, replacing RPA and forming a nucleoprotein filament, which (**D**) initiates the homology search for complementary sequences. (**E**) This prompts DNA strand invasion, where the 3′ end of the invading strand primes DNA synthesis off the sister chromatid. (**F**) After DNA synthesis extends past the DSB, it is precisely repaired.

On the other hand, for DSB repair, the HR pathway provides high fidelity and template-dependent repair of DSBs [94]. DSBs are recognized by the MRN Complex (MRE11-RAD50-NBS1) (Figure 8A), and the DNA ends are captured, leading to the activation of ATM (Ataxia-telangiectasia mutated) [95]. ATM triggers downstream signaling pathways that halt the cell cycle to permit DNA repair (Figure 8B). The MRN complex catalyzes the extensive 5′-3′ end resection to generate single-stranded DNA (ssDNA) overhangs coated by RPA, which protects the DNA against degradation and inhibits the formation of secondary structures (Figure 8B) [96]. Previous studies have shown that ATR (ATM- and Rad3 Related) kinase is recruited via its binding partner ATR-interacting protein (ATRIP) to RPA-covered ssDNA generated at sites of DSBs where ATR is then activated by TopBP1 to phosphorylate downstream targets including the CHK1 signal-transducing kinase [97,98]. RAD51 recombinase displaces RPA from the 3′ overhangs to form presynaptic filaments in a BRCA1-PALB2-BRCA2 dependent manner (Figure 8C). RAD51 forms a nucleoprotein filament that initiates the homology search for complementary sequences on the sister chromatid, called strand invasion (Figure 8D). The invading 3′ end sets up a D-loop intermediate and primes DNA synthesis using the duplex DNA as a template (Figure 8E). After replication extends past the region of DSB, the strand replication continues to the end of the chromosome, and the DSB is precisely repaired (Figure 8F) [95].

Another function of HR repair is to fix DSBs formed during the S phase due to the accumulation of unrepaired SSBs [99]. Poly-ADP-ribose polymerase 1 (PARP1) binds to SSBs and catalyzes the synthesis of additional poly-ADP-ribose (PAR) polymers on target proteins to activate the DNA damage response (DDR) [100]. Once these SSBs are unrepaired and encountered by replication forks, a DSB can occur, which is later repaired through HR [99]. While PARP1 plays a vital role in SSB repair by recruiting XRCC1 and DNA Ligase III, studies have shown that in the absence of PARP1, spontaneous SSBs collapsed replication forks and trigger HR repair [101]. Additional evidence also suggests that in BRCA deficient cells, inhibiting PARP leads to persistent DNA lesions that will no longer be repaired by HR, resulting in synthetic lethality and genomic instability [102]. As it relates to MM, the PARP1 gene is located on chromosome 1q42.12 in an area of frequent amplification in MM [33]. This study reported high PARP1 mRNA expression in GEP-defined poor-risk MM and correlated its expression with shortened disease survival outcomes [33]. Importantly, these mechanistic studies have been proposed to demonstrate that proteasome inhibition with bortezomib induces a functional state of BRCAness in MM cells and sensitizes them to PARP inhibitors’ activity, ultimately resulting in a contextual synthetic lethality [33]. This was also substantiated by Alagpulinsa et al., where the researchers showed that co-treatment of MM cells with Dinaciclib and ABT-888, a cyclin-kinase dependent inhibitor and a PARP1/2 Inhibitor respectively, in vitro resulted in synthetic lethality of MM cells but not normal B cells and slowed the growth of MM xenografts in SCID mice almost by two-fold [103].

Several reports have documented the high-level expression of RAD51 and its paralogs and elevated HR rates in MM cell lines, primary bone marrow aspirates from MM patients, and patient samples compared to normal plasma cells [31,104]. This was also supported by several studies that show high levels of RAD51 expression predict poor event-free and overall survival of MM patients [32]. The inhibition of HR by RAD51 small molecule inhibitors sensitizes cells to doxorubicin [31]. Ultimately, RAD51 appears to be one of the critical proteins that drive HR and targeting it through inhibitor therapy is currently under clinical investigation in various cancers [105].

### 4.4. Regulation of DSB Repair Pathway Choice by 53BP1 and BRCA1

DSB repair pathways choice is critical for maintaining genomic stability [105]. Cell cycle phase and DNA ends can be one of the many factors regulating pathway choice between HR and NHEJ [72]. While HR functions only in the S/G2 phase following DNA replication and requires extensive end processing, NHEJ functions throughout the cell cycle and is only active on minimally processed DNA ends (Figure 6) [106]. Two players, BRCA1 and 53BP1, have been previously implicated in DNA end resection control [77,107].

BRCA1 is an essential player in the DDR that maintains genomic stability and suppresses tumorigenesis by promoting HR repair [108]. 53BP1 is a tumor suppressor protein that promotes NHEJ and suppresses HR [109]. Initially, 53BP1 is recruited to sites of damage where it will interact with a series of players such as ATM, RAP Interacting factor 1 (RIF1), and Pax transactivation domain interacting protein (PTIP) to prevent DNA end resection and promote NHEJ [108]. Alternatively, BRCA1 can counteract 53BP1′s block at DSB ends and promote end resection and HR over NHEJ as a repair pathway [107]. During HR, after ssDNA overhangs are generated through extensive end resection, BRCA1 directly interacts with PALB2 to recruit BRCA2 and RAD51 to DSB sites to prepare for strand invasion.

Germline mutations in factors implicated in DSB repair pathway choice have been investigated in MM patients. One family study examined four MM patients within the same family in three generations. It analyzed the relationship between BRCA1/2 mutations and the appearance of MM by searching for germline mutations in the proband of the family [110]. This study found no deleterious mutation or polymorphism in BRCA1 but found variations in BRCA2 that corresponded with a nonsense mutation and sometimes the loss of 93 amino acids of BRCA2. These results are postulated to be the result of a lack of sufficient sensitive genetic testing methods.

53BP1 is recruited to sites of DSBs is mediated by a direct interaction with histone H4 methylated on lysine 20 (H4K20me) [111]. Previous studies have shown that the induction of DSBs increases methylation of H4K20 that MMSET103 mediates. MMSET is a histone methyltransferase that is often overexpressed in t(4;14) MM patients, believed to be the driving factor in the pathogenesis of this MM subtype [112]. MMSET is one of three NSD family members containing a SET domain and lysine methyltransferase activity. The overexpression of MMSET leads to an increase in proliferation and DNA repair gene expression changes. It has been previously reported to be highly expressed in the gastrointestinal tract and small cell lung carcinomas, tumors of the urinary bladder, female genitals, and skin [113]. In the MM cell line, Shah et al., found that MMSET high cells display enhanced DNA damage repair and increased survival when treated with 0.33 ug/mL bleomycin for one hour [112]. Although some studies have reported that MMSET recruitment to sites of damage can locally induce H4K20 methylation resulting in the recruitment of 53BP1 [114], other studies have not found MMSET-induced H4K20 methylation in vivo and found a different primary target of MMSET (H3K36) [112,115].

## 5. Multiple Myeloma (MM) Treatment

Over the years, researchers have harnessed knowledge about DDR to treat cancer by using treatments such as chemotherapies and radiotherapy that function by inducing DNA damage [17]. DNA damaging agents such as ionizing radiation, topoisomerase inhibitors, bifunctional alkylators, and replication inhibitors are used to induce DSBs [17]. The absence of DDR factors that lead to improper repair or lack thereof positively correlates with therapeutic outcomes [17]. As mentioned earlier, MM is characterized by malignant plasma cells in the bone marrow associated with monoclonal protein in the serum or urine [5,6]. Examination of MM defining events allows for the discrimination between MM and its precursor stages: MGUS and SMM. There are currently numerous combination therapies available for treating MM, such as alkylating agents, corticosteroids, immunomodulatory drugs (IMiDs), proteasome inhibitors (PI), histone deacetylase inhibitors, monoclonal antibody (mAbs) treatment, and autologous stem cell transplant (ASCT) [116]. The treatment of MM depends on age, comorbidities, and previous treatments.

Despite the significant advancements in therapeutics for MM patients, MM remains an incurable disease characterized by multiple remissions and relapses. The current clinical practice focuses on identifying high-risk patients and increasing the quality of life [117,118]. High-risk patients are determined based on many features, including frailty status, aggressiveness in clinical presentation, cytogenic abnormalities, mutations, biochemical abnormalities, prognostic scores, and the ISS risk model [119]. This lack of specificity in defining high-risk features makes it difficult to compare outcomes across the literature. The standard of care for newly diagnosed MM patients of physiological age 70 years or younger who have adequate cardiac pulmonary, hepatic, and renal function is high-dose chemotherapy followed by ASCT [117]. ASCT is not curative but improved median overall survival by around 12 months. Most patients require maintenance following ASCT, such as IMiD Lenalidomide or PI bortezomib for newly diagnosed or high-risk patients, respectively. For patients that are ineligible for ASCT, treatment approaches have been primarily a melphalan and prednisone regimen with modifications based on patient characteristics, including age, performance status, and frailty metrics [120].

In terms of genomic instability and MM features, the International Myeloma Working Group has classified high-risk myeloma by the presence of at least one of the following: del17p or translocations of chromosomes 4, 16, or 20 involving the Ig heavy chain locus: t(4:14) or t(14:20) determined by Fluorescence In Situ Hybridization (FISH).

Melphalan belongs to a class of nitrogen mustard alkylating agents used in the treatment of MM with ASCT [116]. It is an alkylating agent that predominantly induces the monoalkylation of guanine N-7 and adenine in N-3 (90–95%) and ICLs (~5%) between two guanines or guanine and adenine [121,122]. These types of lesions inhibit replication and transcription, leading to mutations and structural changes that induce apoptosis in cells. FA and HR repair the adducts and ICLs induced by melphalan. BER, NHEJ, and NER pathways are also involved in ICL repair. As mentioned throughout this paper, changes in gene activities involved in these pathways or existing polymorphisms can lead to enhanced repair, cell survival, and resistance to melphalan. One study reports that the expression levels of BRCA1, BRCA2, FANCA, FANCC, FANCF, FANCL, and RAD51C were at least increased two-fold in melphalan-resistant MM cell line compared to a parental drug-sensitive cell line [36]. Another study also reported that melphalan resistance in RPMI8226 is characterized by an upregulated LIG4 and XRCC4, proteins involved in NHEJ repair, and downregulated BER glycosylases, NEIL1, UNG2, and MPG [123]. Additionally, one study found that targeting the FA/BRCA pathway by inhibiting NF-kB by siRNA, blocking the IKK complex with BMS-345541, or using bortezomib, a proteasome inhibitor, resulted in diminished FA repair and enhanced melphalan sensitivity [71]. Thus, understanding how DNA repair pathways can confer resistance of MM cells to melphalan presents a potential therapeutic target.

Another alkylating agent, cyclophosphamide, has been used to treat myeloma for over 60 years [106]. Cyclophosphamide has several mechanisms of action that are dose-dependent. It acts as an alkylating agent at high doses, mediating its cytotoxicity through DNA damage. While at low doses, cyclophosphamide has significant immunomodulatory activities that are used to modify the tumor microenvironment and augment existing therapies [124]. Immunomodulation by cyclophosphamide is due to cytotoxic intermediates initiating a complex response that depletes susceptible immune cells, attenuates the function of resilient immune cells, and affects immune function.

Bortezomib is a Proteasome Inhibitor (PI) that reversibly targets the threonine residue of the 26S proteasome, an enzyme that plays a crucial role in regulating protein degradation [125]. The inhibition of the proteasome results in a build-up of regulatory and cell cycle proteins, ultimately leading to cell death. One central mechanism by which bortezomib functions in MM is through stabilizing the NF-kB complex, described as transcriptional activators of the FA pathway [71,126]. Other effects in MM include depleting MM cells of Ubiquitin and abrogation of H2AX polyubiquitination, a necessary step in recruiting BRCA1 and RAD51 to the sites of DSBs and the initiation of HR mediated repair [33]. This bortezomib-induced BRCAness has also been shown to sensitize MM cells to PARP inhibitors, therefore resulting in a contextual synthetic lethality [33]. This introduces a potential clinical benefit of combining bortezomib with PARPi to target DNA repair pathways and induce synthetic lethality.

Immunomodulatory drugs (IMiDs) are structural and functional analogues of thalidomide that are used widely used as induction therapy for both transplant eligible and ineligible patients, in maintenance therapy and for relapsed/refractory disease [127,128]. IMiDs are drugs that can bind to F-box protein cereblon, a member of the Ubiquitin Ligase Complex [129]. Very few data in the literature report a possible association between DNA repair features and IMiDs. One study examined 28 relapsed/refractory MM treated with Thalidomide and found higher response rates in patients with polymorphisms in NHEJ and NER genes: ERCC1, ERCC5, or XRCC5 [130]. They also found more prolonged overall survival in patients with ERCC1 and XRCC5 polymorphisms.

MM patients often experience several serial cycles of response, remission, and relapse during disease progression. As patients experience these relapses, prior therapies become less effective. This reduction in efficacy is driven by the increasing genomic complexity of the tumors and the additional acquisition of a myriad of mutations and epigenetic alterations and highlights the need for new classes of drugs with different mechanisms of action [131].

In summary, based on all the evidence presented in this paper, exploring the role of DNA damage repair pathways in the accumulation of secondary genetic events that contribute to a reduction in efficacy or drug resistance to standard treatments can provide new drug targets that can potentially offer promising results clinically.

## Figures and Tables

**Table 1:** Altered DNA Repair Pathway genes and their effect on MM.

DNA Repair Pathway	Altered Gene in MM	Type of Alteration	Resulting Effect	Reference
**Base Excision Repair**	*OGG1*	SNP	Low BER activity = increased risk of MM progression	[19]
	*MUTYH*	SNP	Shorter survival	[19]
	*APE1*	SNP	Shorter survival	[19]
	*TDG*	Hypermethylation resulting in lower gene expression	less efficient DNA repair in response to OH induced DNA damage	[20]
**Nucleotide Excision Repair**	*hHR23B/RAD23B*	Epigenetic silencing in KAS-6/1	Genetic instability during early B cell maturation	[21]
	*ERCC3* and *XPC*	Knockdown	Impairs NER and increases sensitivity to melphalan	[22]
	*ERR5* and *XPA*	SNP	Longer survival following treatment with autologous stem cell transplantation	[23]
	*XRCC1*	SNP rs25489 (Arg → His)	Diminished repair capacity relevant to MM etiology	[24]
	*ERCC2 N312D* and *ERCC2 K751Q*	Polymorphism resulting in an amino acid substitution	Increased DNA adducts, low repair capacity associated with sensitivity to high dose alkylating chemotherapy	[25]
**Mismatch Repair**	*hMLH1*	Hypermethylation associated with decreased protein expression but not loss of MMR capacity	Plays a role in the transition of MGUS to MM and poorer survival	[26,27]
	*MSI*	Deficiencies in MMR repair proteins	Possible contributory role in MM disease progression	[28]
**Homologous Recombination**	*ATM* and *BRCA2*	Deleterious mutations	Negative impact on survival	[29]
	*ATM*	Mutations	Impaired progression-free survival, Impaired overall survival	[30]
	*RAD51*	High expression	Chemoresistance and poor patient survival	[31]
	*RAD51*	DNA Repair Score	Poor event free and overall survival	[32]
	*PARP1*	Higher mRNA expression	Poor survival in MM	[33]
**Non-Homologous End Joining**	*LIG4*	SNP A3V and T9I resulting in amino acid substitution	Two-fold reduction in risk of developing MM	[34]
	*XRCC4*	SNP affecting mRNA transcript or altering expression	Alters the risk of developing myeloma	[35]
	*XRCC5*	SNP causing errors in alternative splicing, affect mRNA stability and translation		
**Fanconi Anaemia**	*FANCF* and *RAD51C*	Increase in expression of genes	Acquired Melphalan resistance in 8226/LR5	[36]
	*NEIL1*	Downregulation	Increased efficiency of DSB and ss break repair associated with Melphalan resistance	[37]
	*FANCI, FANCA, FANCD2,* and *BRCA*	Alterations such as Missense Mutations	Related to MM patients in relapse	[38]
	*FANCI, FANB, RAD51*, and *BRCA1*	Increased expression	Characteristics of residual myeloma plasma cells (cells responsible for relapse, drug resistance, and having stem cell-like characteristics including altered cell metabolism, increased drug efflux, ALDH1 activity, and propagation)	[39]
**Overlapping pathways**	*MMSET*	Overexpression	Poorer prognosis and response to therapy compared to other MM subtypes	[40]
	*AID*	Absent in MM and SMM patient samples	Early and common driver mutational process	[41]

## Data Availability

Not applicable.

## References

[1] Bird S.A., Boyd K. (2019). Multiple myeloma: An overview of management. Palliat. Care Soc. Pract..

[2] Fairfield H., Falank C., Avery L., Reagan M.R. (2016). Multiple myeloma in the marrow: Pathogenesis and treatments. Ann. N. Y. Acad. Sci..

[3] Ludwig H., Novis Durie S., Meckl A., Hinke A., Durie B. (2020). Multiple Myeloma Incidence and Mortality Around the Globe; Interrelations Between Health Access and Quality, Economic Resources, and Patient Empowerment. Oncologist.

[4] Albagoush S.A., Azevedo A.M. (2021). Multiple Myeloma.

[5] Bergstrom D.J., Kotb R., Louzada M.L., Sutherland H.J., Tavoularis S., Venner C.P., Côté J., LeBlanc R., Reiman A., Sebag M. (2020). Consensus Guidelines on the Diagnosis of Multiple Myeloma and Related Disorders: Recommendations of the Myeloma Canada Research Network Consensus Guideline Consortium. Clin. Lymphoma Myeloma Leuk..

[6] Durie B.G., Salmon S.E. (1975). A clinical staging system for multiple myeloma. Correlation of measured myeloma cell mass with presenting clinical features, response to treatment, and survival. Cancer.

[7] Willrich M.A., Katzmann J.A. (2016). Laboratory testing requirements for diagnosis and follow-up of multiple myeloma and related plasma cell dyscrasias. Clin. Chem. Lab. Med..

[8] Janz S., Zhan F., Sun F., Cheng Y., Pisano M., Yang Y., Goldschmidt H., Hari P. (2019). Germline Risk Contribution to Genomic Instability in Multiple Myeloma. Front. Genet..

[9] Dewald G.W., Kyle R.A., Hicks G.A., Greipp P.R. (1985). The clinical significance of cytogenetic studies in 100 patients with multiple myeloma, plasma cell leukemia, or amyloidosis. Blood.

[10] Constantinescu A., Schlissel M.S. (1997). Changes in locus-specific V(D)J recombinase activity induced by immunoglobulin gene products during B cell development. J. Exp. Med..

[11] Dunnick W.A., Collins J.T., Shi J., Westfield G., Fontaine C., Hakimpour P., Papavasiliou F.N. (2009). Switch recombination and somatic hypermutation are controlled by the heavy chain 3’ enhancer region. J. Exp. Med..

[12] Alagpulinsa D.A., Szalat R.E., Poznansky Mark C., Shmookler Reis R.J. (2020). Genomic Instability in Multiple Myeloma. Trends Cancer Res..

[13] Bergsagel P.L., Kuehl W.M. (2001). Chromosome translocations in multiple myeloma. Oncogene.

[14] Thapa B., Awada H., Dong J., Gurnari C., Hari P., Dhakal B. (2021). A Comprehensive Review of the Genomics of Multiple Myeloma: Evolutionary Trajectories, Gene Expression Profiling, and Emerging Therapeutics. Cells.

[15] Lawasut P., Groen R.W., Dhimolea E., Richardson P.G., Anderson K.C., Mitsiades C.S. (2013). Decoding the pathophysiology and the genetics of multiple myeloma to identify new therapeutic targets. Semin. Oncol..

[16] Alhmoud J.F., Woolley J.F., Al Moustafa A.-E., Malki M.I. (2020). DNA Damage/Repair Management in Cancers. Cancers.

[17] Jackson S.P., Bartek J. (2009). The DNA-damage response in human biology and disease. Nature.

[18] Srinivas U.S., Tan B.W.Q., Vellayappan B.A., Jeyasekharan A.D. (2019). ROS and the DNA damage response in cancer. Redox Biol..

[19] Ushie C., Saitoh T., Iwasaki A., Moriyama N., Hattori H., Matsumoto M., Sawamura M., Isoda J., Handa H., Yokohama A. (2012). The Polymorphisms of Base Excision Repair Genes Influence the Prognosis of Multiple Myeloma. Blood.

[20] Peng B., Hurt E.M., Hodge D.R., Thomas S.B., Farrar W.L. (2006). DNA hypermethylation and partial gene silencing of human thymine—DNA glycosylase in multiple myeloma cell lines. Epigenetics.

[21] Peng B., Hodge D.R., Thomas S.B., Cherry J.M., Munroe D.J., Pompeia C., Xiao W., Farrar W.L. (2005). Epigenetic silencing of the human nucleotide excision repair gene, hHR23B, in interleukin-6-responsive multiple myeloma KAS-6/1 cells. J. Biol. Chem..

[22] Szalat R., Samur M.K., Fulciniti M., Lopez M., Nanjappa P., Cleynen A., Wen K., Kumar S., Perini T., Calkins A.S. (2018). Nucleotide excision repair is a potential therapeutic target in multiple myeloma. Leukemia.

[23] De Larrea C.F., Navarro A., Tovar N., Pedrosa F., Díaz T., Cibeira M.T., Magnano L., Rosiñol L., Rovira M., Rozman M. (2012). Impact of Single Nucleotide Polymorphisms in Genes Involved in DNA Repair and Drug Metabolism On Survival After Autologous Stem Cell Transplantation in Patients with Multiple Myeloma. Blood.

[24] Rand K.A., Conti D.V., Haiman C.A., Van Den Berg D.J., Birmann B.M., De Roos A.J., Severson R.K., Gebregziabher M., Ailawadhi S., Morbacher A. (2012). Abstract 2634: Polymorphisms in DNA repair genes and risk of multiple myeloma. Cancer Res..

[25] Vangsted A., Gimsing P., Klausen T.W., Nexø B.A., Wallin H., Andersen P., Hokland P., Lillevang S.T., Vogel U. (2007). Polymorphisms in the genes ERCC2, XRCC3 and CD3EAP influence treatment outcome in multiple myeloma patients undergoing autologous bone marrow transplantation. Int. J. Cancer.

[26] Martin P., Garcia-Cosio M., Santon A., Bellas C. (2008). Aberrant gene promoter methylation in plasma cell dyscrasias. Exp. Mol. Pathol..

[27] Martin P., Santón A., García-Cosio M., Bellas C. (2006). hMLH1 and MGMT inactivation as a mechanism of tumorigenesis in monoclonal gammopathies. Mod. Pathol..

[28] Velangi M.R., Matheson E.C., Morgan Gareth J., Jackson G.H., Taylor P.R., Hall Andrew G., Irving J.A.E. (2004). DNA mismatch repair pathway defects in the pathogenesis and evolution of myeloma. Carcinogenesis.

[29] Pawlyn C., Loehr A., Ashby C., Tytarenko R., Deshpande S., Sun J., Fedorchak K., Mughal T., Davies F.E., Walker B.A. (2018). Loss of heterozygosity as a marker of homologous repair deficiency in multiple myeloma: A role for PARP inhibition?. Leukemia.

[30] Walker B.A., Boyle E.M., Wardell C.P., Murison A., Begum D.B., Dahir N.B., Proszek P.Z., Johnson D.C., Kaiser M.F., Melchor L. (2015). Mutational Spectrum, Copy Number Changes, and Outcome: Results of a Sequencing Study of Patients With Newly Diagnosed Myeloma. J. Clin. Oncol..

[31] Alagpulinsa D.A., Ayyadevara S., Shmookler Reis R.J. (2014). A Small-Molecule Inhibitor of RAD51 Reduces Homologous Recombination and Sensitizes Multiple Myeloma Cells to Doxorubicin. Front. Oncol..

[32] Kassambara A., Gourzones-Dmitriev C., Sahota S., Rème T., Moreaux J., Goldschmidt H., Constantinou A., Pasero P., Hose D., Klein B. (2014). A DNA repair pathway score predicts survival in human multiple myeloma: The potential for therapeutic strategy. Oncotarget.

[33] Neri P., Ren L., Gratton K., Stebner E., Johnson J., Klimowicz A., Duggan P., Tassone P., Mansoor A., Stewart D.A. (2011). Bortezomib-induced “BRCAness’’ sensitizes multiple myeloma cells to PARP inhibitors. Blood.

[34] Roddam P.L., Rollinson S., O’Driscoll M., Jeggo P.A., Jack A., Morgan G.J. (2002). Genetic variants of NHEJ DNA ligase IV can affect the risk of developing multiple myeloma, a tumour characterised by aberrant class switch recombination. J. Med. Genet..

[35] Hayden P.J., Tewari P., Morris D.W., Staines A., Crowley D., Nieters A., Becker N., de Sanjosé S., Foretova L., Maynadié M. (2007). Variation in DNA repair genes XRCC3, XRCC4, XRCC5 and susceptibility to myeloma. Hum. Mol. Genet..

[36] Chen Q., der Sluis P.C., Boulware D., Hazlehurst L.A., Dalton W.S. (2005). The FA/BRCA pathway is involved in melphalan-induced DNA interstrand cross-link repair and accounts for melphalan resistance in multiple myeloma cells. Blood.

[37] Neri P., Bahlis N.J. (2013). Genomic instability in multiple myeloma: Mechanisms and therapeutic implications. Expert Opin. Biol. Ther..

[38] Jacquemont C., Taniguchi T. (2007). Proteasome Function Is Required for DNA Damage Response and Fanconi Anemia Pathway Activation. Cancer Res..

[39] Bauer M.A., Henry M., Patel P., Wang Y., Epstein J., Davies F.E., Schinke C.D., Zangari M., Thanendrarajan S., van Rhee F. (2018). Expression Signature of Myeloma Residual Cells Is Characterized By Genes Associated with Proliferation, Epigenetic Modification, and Stem Cell Maintenance. Blood.

[40] Keats J.J., Reiman T., Maxwell C.A., Taylor B.J., Larratt L.M., Mant M.J., Belch A.R., Pilarski L.M. (2003). In multiple myeloma, t (4;14)(p16;q32) is an adverse prognostic factor irrespective of FGFR3 expression. Blood.

[41] Bolli N., Maura F., Minvielle S., Gloznik D., Szalat R., Fullam A., Martincorena I., Dawson K.J., Samur M.K., Zamora J. (2018). Genomic patterns of progression in smoldering multiple myeloma. Nat. Commun..

[42] Stratigopoulou M., van Dam T.P., Guikema J.E.J. (2020). Base Excision Repair in the Immune System: Small DNA Lesions With Big Consequences. Front. Immunol..

[43] Krokan H.E., Bjørås M. (2013). Base excision repair. Cold Spring Harb. Perspect. Biol..

[44] Jacobs A.L., Schär P. (2012). DNA glycosylases: In DNA repair and beyond. Chromosoma.

[45] Whitaker A.M., Schaich M.A., Smith M.R., Flynn T.S., Freudenthal B.D. (2017). Base excision repair of oxidative DNA damage: From mechanism to disease. Front. Biosci..

[46] Weissman L., Jo D.G., Sørensen M.M., de Souza-Pinto N.C., Markesbery W.R., Mattson M.P., Bohr V.A. (2007). Defective DNA base excision repair in brain from individuals with Alzheimer’s disease and amnestic mild cognitive impairment. Nucleic Acids Res..

[47] Goula A.-V., Merienne K. (2013). Abnormal base excision repair at trinucleotide repeats associated with diseases: A tissue-selective mechanism. Genes.

[48] Ba X., Aguilera-Aguirre L., Sur S., Boldogh I. (2015). 8-Oxoguanine DNA glycosylase-1-driven DNA base excision repair: Role in asthma pathogenesis. Curr. Opin. Allergy Clin. Immunol..

[49] Simon R., Meller R., Yang T., Pearson A., Wilson G. (2019). Enhancing Base Excision Repair of Mitochondrial DNA to Reduce Ischemic Injury Following Reperfusion. Transl. Stroke Res..

[50] Kusakabe M., Onishi Y., Tada H., Kurihara F., Kusao K., Furukawa M., Iwai S., Yokoi M., Sakai W., Sugasawa K. (2019). Mechanism and regulation of DNA damage recognition in nucleotide excision repair. Genes Environ..

[51] Marteijn J.A., Lans H., Vermeulen W., Hoeijmakers J.H.J. (2014). Understanding nucleotide excision repair and its roles in cancer and ageing. Nat. Rev. Mol. Cell Biol..

[52] Hsieh P., Yamane K. (2008). DNA mismatch repair: Molecular mechanism, cancer, and ageing. Mech. Ageing Dev..

[53] Huang Y., Li G.-M. (2018). DNA mismatch repair preferentially safeguards actively transcribed genes. DNA Repair.

[54] Liu D., Keijzers G., Rasmussen L.J. (2017). DNA mismatch repair and its many roles in eukaryotic cells. Mutat. Res. Rev. Mutat. Res..

[55] Vilar E., Gruber S.B. (2010). Microsatellite instability in colorectal cancer-the stable evidence. Nat. Rev. Clin. Oncol..

[56] Yang W. (2000). Structure and function of mismatch repair proteins. Mutat. Res..

[57] Kunkel T.A., Erie D.A. (2005). DNA mismatch repair. Annu. Rev. Biochem..

[58] Kadyrov F.A., Dzantiev L., Constantin N., Modrich P. (2006). Endonucleolytic function of MutLalpha in human mismatch repair. Cell.

[59] Kadyrova L.Y., Gujar V., Burdett V., Modrich P.L., Kadyrov F.A. (2020). Human MutLγ, the MLH1-MLH3 heterodimer, is an endonuclease that promotes DNA expansion. Proc. Natl. Acad. Sci. USA.

[60] Miller C.J., Kim G.-Y., Zhao X., Usdin K. (2020). All three mammalian MutL complexes are required for repeat expansion in a mouse cell model of the Fragile X-related disorders. PLoS Genet..

[61] Timurağaoğlu A., Demircin S., Dizlek S., Alanoğlu G., Kiriş E. (2009). Microsatellite instability is a common finding in multiple myeloma. Clin. Lymphoma Myeloma.

[62] Gu X., Shivarov V., Strout M.P. (2012). The role of activation-induced cytidine deaminase in lymphomagenesis. Curr. Opin. Hematol..

[63] Pasqualucci L., Guglielmino R., Houldsworth J., Mohr J., Aoufouchi S., Polakiewicz R., Chaganti R.S.K., Dalla-Favera R. (2004). Expression of the AID protein in normal and neoplastic B cells. Blood.

[64] Ceccaldi R., Sarangi P., D’Andrea A.D. (2016). The Fanconi anaemia pathway: New players and new functions. Nat. Rev. Mol. Cell Biol..

[65] Liu W., Palovcak A., Li F., Zafar A., Yuan F., Zhang Y. (2020). Fanconi anemia pathway as a prospective target for cancer intervention. Cell Biosci..

[66] Rodríguez A., D’Andrea A. (2017). Fanconi anemia pathway. Curr. Biol..

[67] Kutler D.I., Singh B., Satagopan J., Batish S.D., Berwick M., Giampietro P.F., Hanenberg H., Auerbach A.D. (2003). A 20-year perspective on the International Fanconi Anemia Registry (IFAR). Blood.

[68] Garner E., Smogorzewska A. (2011). Ubiquitylation and the Fanconi anemia pathway. FEBS Lett..

[69] Chauhan D., Ajita D., Singh D., Anderson K., Lonial S. (2008). Proteasome Inhibitors as Therapy in Multiple Myeloma. Myeloma Therapy: Pursuing the Plasma Cell.

[70] Mitsiades N., Mitsiades C.S., Richardson P.G., Poulaki V., Tai Y.T., Chauhan D., Fanourakis G., Gu X., Bailey C., Joseph M. (2003). The proteasome inhibitor PS-341 potentiates sensitivity of multiple myeloma cells to conventional chemotherapeutic agents: Therapeutic applications. Blood.

[71] Yarde D.N., Oliveira V., Mathews L., Wang X., Villagra A., Boulware D., Shain K.H., Hazlehurst L.A., Alsina M., Chen D.T. (2009). Targeting the Fanconi anemia/BRCA pathway circumvents drug resistance in multiple myeloma. Cancer Res..

[72] Brandsma I., Gent D.C. (2012). Pathway choice in DNA double strand break repair: Observations of a balancing act. Genome Integr..

[73] Yang C., Betti C., Singh S., Toor A., Vaughan A. (2009). Impaired NHEJ function in multiple myeloma. Mutat. Res..

[74] Setton J., Bindra R.S., Powell S.N. (2016). DNA double-strand repair by nonhomologous end joining and its clinical relevance. DNA Repair in Cancer Therapy: Molecular Targets and Clinical Applications.

[75] Roth D.B. (2014). V(D)J Recombination: Mechanism, Errors, and Fidelity. Microbiol. Spectr..

[76] Chang H.H.Y., Pannunzio N.R., Adachi N., Lieber M.R. (2017). Non-homologous DNA end joining and alternative pathways to double-strand break repair. Nat. Rev. Mol. Cell Biol..

[77] Daley J.M., Sung P. (2014). 53BP1, BRCA1, and the Choice between Recombination and End Joining at DNA Double-Strand Breaks. Mol. Cell. Biol..

[78] Pannunzio N.R., Li S., Watanabe G., Lieber M.R. (2014). Non-homologous end joining often uses microhomology: Implications for alternative end joining. DNA Repair.

[79] Kang Y.J., Yan C.T. (2018). Regulation of DNA repair in the absence of classical non-homologous end joining. DNA Repair.

[80] Chiruvella K.K., Liang Z., Wilson T.E. (2013). Repair of double-strand breaks by end joining. Cold Spring Harb. Perspect. Biol..

[81] Gassner F.J., Schubert M., Rebhandl S., Spandl K., Zaborsky N., Catakovic K., Blaimer S., Hebenstreit D., Greil R., Geisberger R. (2018). Imprecision and DNA Break Repair Biased towards Incompatible End Joining in Leukemia. Mol. Cancer Res..

[82] Sallmyr A., Tomkinson A.E. (2018). Repair of DNA double-strand breaks by mammalian alternative end-joining pathways. J. Biol. Chem..

[83] Seol J.-H., Shim E.Y., Lee S.E. (2018). Microhomology-mediated end joining: Good, bad and ugly. Mutat. Res..

[84] Herrero A.B., San Miguel J., Gutierrez N.C. (2015). Deregulation of DNA double-strand break repair in multiple myeloma: Implications for genome stability. PLoS ONE.

[85] Campa D., Martino A., Macauda A., Dudziński M., Suska A., Druzd-Sitek A., Raab M.S., Moreno V., Huhn S., Butrym A. (2019). Genetic polymorphisms in genes of class switch recombination and multiple myeloma risk and survival: An IMMEnSE study. Leuk. Lymphoma.

[86] Malu S., Malshetty V., Francis D., Cortes P. (2012). Role of non-homologous end joining in V(D)J recombination. Immunol. Res..

[87] Rooney S., Alt F.W., Sekiguchi J., Manis J.P. (2005). Artemis-independent functions of DNA-dependent protein kinase in Ig heavy chain class switch recombination and development. Proc. Natl. Acad. Sci. USA.

[88] Crowe J.L., Wang X.S., Shao Z., Lee B.J., Estes V.M., Zha S. (2020). DNA-PKcs phosphorylation at the T2609 cluster alters the repair pathway choice during immunoglobulin class switch recombination. Proc. Natl. Acad. Sci. USA.

[89] Pierce A.J., Jasin M. (2001). NHEJ deficiency and disease. Mol. Cell.

[90] Difilippantonio M.J., Zhu J., Chen H.T., Meffre E., Nussenzweig M.C., Max E.E., Ried T., Nussenzweig A. (2000). DNA repair protein Ku80 suppresses chromosomal aberrations and malignant transformation. Nature.

[91] Gu Y., Jin S., Gao Y., Weaver D.T., Alt F.W. (1997). Ku70-deficient embryonic stem cells have increased ionizing radiosensitivity, defective DNA end-binding activity, and inability to support V(D)J recombination. Proc. Natl. Acad. Sci. USA.

[92] Taylor B.J., Pittman J.A., Belch A.R., Pilarski L.M. (2004). VDJ-Switch Region Analysis in Multiple Myeloma Patients Reveals Homogeneity and Long-Term Stability of Switch Junctions, and Ongoing Mutation Upstream of Switch Mu. Blood.

[93] Bergsagel P.L., Kuehl W.M., Rowley J.D., le Beau M.M., Rabbitts T.H. (2015). Immunoglobulin and MYC Rearrangements in Multiple Myeloma Pathogenesis. Chromosomal Translocations and Genome Rearrangements in Cancer.

[94] Li X., Heyer W.-D. (2008). Homologous recombination in DNA repair and DNA damage tolerance. Cell Res..

[95] Chapman J.R., Taylor M.R.G., Boulton S.J. (2012). Playing the end game: DNA double-strand break repair pathway choice. Mol. Cell.

[96] Alani E., Thresher R., Griffith J.D., Kolodner R.D. (1992). Characterization of DNA-binding and strand-exchange stimulation properties of y-RPA, a yeast single-strand-DNA-binding protein. J. Mol. Biol..

[97] Kumagai A., Lee J., Yoo H.Y., Dunphy W.G. (2006). TopBP1 activates the ATR-ATRIP complex. Cell.

[98] Mordes D.A., Glick G.G., Zhao R., Cortez D. (2008). TopBP1 activates ATR through ATRIP and a PIKK regulatory domain. Genes Dev..

[99] Metzger M.J., Stoddard B.L., Monnat R.J. (2013). PARP-mediated repair, homologous recombination, and back-up non-homologous end joining-like repair of single-strand nicks. DNA Repair.

[100] Ray Chaudhuri A., Nussenzweig A. (2017). The multifaceted roles of PARP1 in DNA repair and chromatin remodelling. Nat. Rev. Mol. Cell Biol..

[101] Bryant H.E., Schultz N., Thomas H.D., Parker K.M., Flower D., Lopez E., Kyle S., Meuth M., Curtin N.J., Helleday T. (2005). Specific killing of BRCA2-deficient tumours with inhibitors of poly(ADP-ribose) polymerase. Nature.

[102] Farmer H., McCabe N., Lord C.J., Tutt A.N., Johnson D.A., Richardson T.B., Santarosa M., Dillon K.J., Hickson I., Knights C. (2005). Targeting the DNA repair defect in BRCA mutant cells as a therapeutic strategy. Nature.

[103] Alagpulinsa D.A., Ayyadevara S., Yaccoby S., Shmookler Reis R.J. (2016). A Cyclin-Dependent Kinase Inhibitor, Dinaciclib, Impairs Homologous Recombination and Sensitizes Multiple Myeloma Cells to PARP Inhibition. Mol. Cancer Ther..

[104] Shammas M.A., Shmookler Reis R.J., Koley H., Batchu R.B., Li C., Munshi N.C. (2009). Dysfunctional homologous recombination mediates genomic instability and progression in myeloma. Blood.

[105] Case Medical Research (2019). A phase 1/2 study of CYT-0851, an oral RAD51 inhibitor. B-Cell Malignancies and Advanced Solid Tumors.

[106] Swan D., Gurney M., Krawczyk J., Ryan A., O’Dwyer M. (2020). Beyond DNA Damage: Exploring the Immunomodulatory Effects of Cyclophosphamide in Multiple Myeloma. HemaSphere.

[107] Zhao F., Kim W., Kloeber J.A., Lou Z. (2020). DNA end resection and its role in DNA replication and DSB repair choice in mammalian cells. Exp. Mol. Med..

[108] Liu Y., Lu L.-Y. (2020). BRCA1 and homologous recombination: Implications from mouse embryonic development. Cell Biosci..

[109] Mirza-Aghazadeh-Attari M., Mohammadzadeh A., Yousefi B., Mihanfar A., Mihanfar A., Majidinia M. (2019). 53BP1: A key player of DNA damage response with critical functions in cancer. DNA Repair.

[110] Sobol H., Vey N., Sauvan R., Philip N., Noguchi T., Eisinger F. (2002). Re: Familial Multiple Myeloma: A Family Study and Review of the Literature. J. Natl. Cancer Inst..

[111] Botuyan M.V., Lee J., Ward I.M., Kim J.E., Thompson J.R., Chen J., Mer G. (2006). Structural Basis for the Methylation State-Specific Recognition of Histone H4-K20 by 53BP1 and Crb2 in DNA Repair. Cell.

[112] Shah M.Y., Martinez-Garcia E., Phillip J.M., Chambliss A.B., Popovic R., Ezponda T., Small E.C., Will C., Phillip M.P., Neri P. (2016). MMSET/WHSC1 enhances DNA damage repair leading to an increase in resistance to chemotherapeutic agents. Oncogene.

[113] Hudlebusch H.R., Santoni-Rugiu E., Simon R., Ralfkiær E., Rossing H.H., Johansen J.V., Jørgensen M., Sauter G., Helin K. (2011). The histone methyltransferase and putative oncoprotein MMSET is overexpressed in a large variety of human tumors. Clin. Cancer Res..

[114] Pei H., Zhang L., Luo K., Qin Y., Chesi M., Fei F., Bergsagel P.L., Wang L., You Z., Lou Z. (2011). MMSET regulates histone H4K20 methylation and 53BP1 accumulation at DNA damage sites. Nature.

[115] Martinez-Garcia E., Popovic R., Min D.J., Sweet S.M., Thomas P.M., Zamdborg L., Heffner A., Will C., Lamy L., Staudt L.M. (2011). The MMSET histone methyl transferase switches global histone methylation and alters gene expression in t (4;14) multiple myeloma cells. Blood.

[116] Poczta A., Rogalska A., Marczak A. (2021). Treatment of Multiple Myeloma and the Role of Melphalan in the Era of Modern Therapies-Current Research and Clinical Approaches. J. Clin. Med. Res..

[117] Rajkumar S.V., Kumar S. (2020). Multiple myeloma current treatment algorithms. Blood Cancer J..

[118] Cazaubiel T., Mulas O., Montes L., Schavgoulidze A., Avet-Loiseau H., Corre J., Perrot A. (2020). Risk and Response-Adapted Treatment in Multiple Myeloma. Cancers.

[119] Usmani S., Rodriguez-Otero P., Bhutani M., Mateos M.V., Miguel J.S. (2015). Defining and treating high-risk multiple myeloma. Leukemia.

[120] Kumar S.K., Rajkumar V., Kyle R.A., van Duin M., Sonneveld P., Mateos M.-V., Gay F., Anderson K.C. (2017). Multiple myeloma. Nat. Rev. Dis. Primers.

[121] Muniandy P.A., Liu J., Majumdar A., Liu S., Seidman M.M. (2010). DNA interstrand crosslink repair in mammalian cells: Step by step. Crit. Rev. Biochem. Mol. Biol..

[122] Osborne M.R., Wilman D.E., Lawley P.D. (1995). Alkylation of DNA by the Nitrogen Mustard Bis-(2-chloroethyl) methylamine. Chem. Res. Toxicol..

[123] Sousa M.M.L., Zub K.A., Aas P.A., Hanssen-Bauer A., Demirovic A. (2013). An Inverse Switch in DNA Base Excision and Strand Break Repair Contributes to Melphalan Resistance in Multiple Myeloma Cells. PLoS ONE.

[124] Madondo M.T., Quinn M., Plebanski M. (2016). Low dose cyclophosphamide: Mechanisms of T cell modulation. Cancer Treat. Rev..

[125] Field-Smith A., Morgan G.J., Davies F.E. (2006). Bortezomib (Velcade) in the Treatment of Multiple Myeloma. Ther. Clin. Risk Manag..

[126] Hideshima T., Ikeda H., Chauhan D., Okawa Y., Raje N., Podar K., Mitsiades C., Munshi N.C., Richardson P.G., Carrasco R.D. (2009). Bortezomib induces canonical nuclear factor-κB activation in multiple myeloma cells. Blood.

[127] Holstein S.A., Mccarthy P.L. (2017). Immunomodulatory Drugs in Multiple Myeloma: Mechanisms of Action and Clinical Experience. Drugs.

[128] Quach H., Ritchie D., Stewart A.K., Neeson P., Harrison S., Smyth M.J., Prince H.M. (2010). Mechanism of action of immunomodulatory drugs (IMiDS) in multiple myeloma. Leukemia.

[129] Takumi I., Ando H., Suzuki T., Ogura T., Hotta K., Imamura Y., Yamaguchi Y., Handa H. (2010). Identification of a Primary Target of Thalidomide Teratogenicity. Science.

[130] Cibeira M.T., de Larrea C.F., Navarro A., Díaz T., Fuster D., Tovar N., Rosiñol L., Monzó M., Bladé J. (2011). Impact on response and survival of DNA repair single nucleotide polymorphisms in relapsed or refractory multiple myeloma patients treated with thalidomide. Leukemia Res..

[131] Keats J.J., Chesi M., Egan J.B., Garbitt V.M., Palmer S.E., Braggio E., Van Wier S., Blackburn P.R., Baker A.S., Dispenzieri A. (2012). Clonal competition with alternating dominance in multiple myeloma. Blood.

